# Magnetic Hydrogel Beads as a Reusable Adsorbent for Highly Efficient and Rapid Removal of Aluminum: Characterization, Response Surface Methodology Optimization, and Evaluation of Isotherms, Kinetics, and Thermodynamic Studies

**DOI:** 10.1021/acsomega.3c04984

**Published:** 2023-11-03

**Authors:** Raif İlktaç, Ece Bayir

**Affiliations:** Ege University Central Research Test and Analysis Laboratory Application and Research Center (EGE-MATAL), Izmir 35100, Turkey

## Abstract

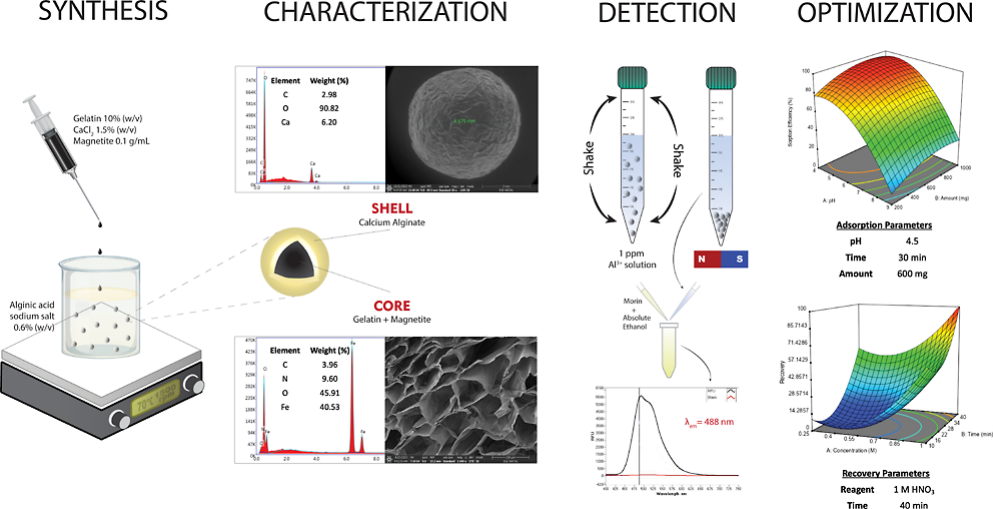

Biopolymers such as alginate and gelatin have attracted much attention because of their exceptional adsorption properties and biocompatibility. The magnetic hydrogel beads produced and used in this study had a core structure composed of magnetite nanoparticles and gelatin and a shell structure composed of alginate. The combination of the metal-ion binding ability of alginate and the mechanical strength of gelatin in magnetic hydrogel beads presents a new approach for the removal of metal from water sources. The beads were designed for aluminum removal and fully characterized using various methods, including Fourier transform infrared spectroscopy, X-ray photoelectron spectroscopy, scanning electron microscopy–energy-dispersive X-ray spectroscopy, vibrating sample magnetometry, microcomputed tomography, and dynamic mechanical analysis. Statistical experimental designs were employed to optimize the parameters of the adsorption and recovery processes. Plackett–Burman Design, Box–Behnken Design, and Central Composite Design were used for identifying the significant factors and optimizing the parameters of the adsorption and recovery processes, respectively. The optimum parameters determined for adsorption are as follows: pH: 4, contact time: 30 min, adsorbent amount: 600 mg; recovery time: reagent 1 M HNO_3_; and contact time: 40 min. The adsorption process was described by using the Langmuir isotherm model. It reveals a homogeneous bead surface and monolayer adsorption with an adsorption capacity of 5.25 mg g^–1^. Limit of detection and limit of quantification values were calculated as 4.3 and 14 μg L^–1^, respectively. The adsorption process was described by a pseudo-second-order kinetic model, which assumes that chemisorption is the rate-controlling mechanism. Thermodynamic studies indicate that adsorption is spontaneous and endothermic. The adsorbent was reusable for 10 successive adsorption–desorption cycles with a quantitative adsorption of 98.2% ± 0.3% and a recovery of 99.4% ± 2.6%. The minimum adsorbent dose was determined as 30 g L^–1^ to achieve quantitative adsorption of aluminum. The effects of the inorganic ions were also investigated. The proposed method was applied to tap water and carboy water samples, and the results indicate that magnetic hydrogel beads can be an effective and reusable bioadsorbent for the detection and removal of aluminum in water samples. The recovery values obtained by using the developed method were quantitative and consistent with the results obtained from the inductively coupled plasma optical emission spectrometer.

## 1 Introduction

Aluminum is a versatile metal commonly used in many industrial sectors, especially in construction, packaging, and transportation.^1,2^ It is abundant, recyclable, corrosion resistant, and thermally and electrically conductive, making it a valuable material for these sectors. However, overexposure to aluminum ions or compounds may result in adverse effects, such as Alzheimer’s disease, dementia, anemia, and bone disorders.^3−6^ Thus, it is important to identify the presence of aluminum in various commonly used materials to ensure their safe handling. In this regard, the World Health Organization standard for the maximum permissible level of aluminum in drinking water is 200 μg/L.^7^ Different spectroscopic and electrochemical methods have been widely used for aluminum detection.^8−12^ Prior to the detection of trace levels of metal ions, the usage of adsorbents can eliminate or minimize the sample matrix effects and improve the sensitivity of the method.^13^ Various types of adsorbents have been used for the removal and detection of metal ions in environmental, biological, and food samples.^14,15^ Especially in the past decade, adsorbents derived from natural materials such as chitosan, cellulose, carrageenan, and alginate have been widely used for the removal of diverse metal ions.^16−19^ Different types of self-assembled gel structures have also been employed in several fields, such as biomedical research, tissue engineering, and drug delivery systems for removal of contaminants.^20−22^

Alginate is a biocompatible, biodegradable, and nontoxic natural polymer derived from brown seaweed. Its functional groups provide excellent adsorption properties for the removal of contaminants, wastewater treatment, and environmental remediation.^23^ Its gel-forming properties further enhance its adsorption capabilities. Alginate hydrogel beads or membranes can be prepared by ionotropic gelation, a process in which alginate is cross-linked with divalent cations such as calcium ions.^24^ Gelatin, a biodegradable biopolymer derived from the denatured collagen of animals, possesses several desirable characteristics that make it an attractive adsorbent material.^25^ Alginate–gelatin composite adsorbents have emerged as a promising class of materials for metal-ion adsorption because of their synergistic properties and enhanced adsorption capabilities. Alginate provides excellent metal-ion binding properties, while gelatin contributes to the mechanical strength and stability of the composite.^26^ Gelatin enhances the structural integrity of the adsorbent, prevents disintegration, and maintains its performance during the adsorption process. Hydrogel structures have large surface areas and porosities, enabling efficient adsorption. However, they typically exhibit poor mechanical properties, owing to their highly swollen network structure, resulting in limited reusability. Therefore, researchers have endeavored to enhance the mechanical properties of the hydrogels used in adsorption and desorption studies. One way to enhance the mechanical strength and stability of these hydrogels is through cross-linking with glutaraldehyde (GA), resulting in a three-dimensional (3D) network structure that reinforces the hydrogel matrix and enhances its mechanical properties.^27^ Stability enhancement facilitates the repeated use of the adsorbent, reduces operational costs, and minimizes waste generation. To reuse the adsorbent after sorption, the beads must be filtered out of the solution, which is a time-consuming process. To accelerate the separation process, magnetic particles were encapsulated within the alginate–gelatin beads. The acquired magnetism of the adsorbent enabled rapid separation from the solution by using a magnet. In this study, magnetic hydrogel beads were used as an adsorbent in the analysis of aluminum, and statistical approaches were employed for the experimental design and optimization of adsorption and desorption processes.

Plackett–Burman design (PBD) is a statistical experimental design that has a wide range of applications in various domains including chemical engineering, materials science, and environmental science. This design is particularly useful for screening a large number of factors by employing a relatively small number of experiments and identifying the significant factors that have the greatest impact on the process.^28^ In this study, PBD was used to determine the most significant factors from five numerical and two categorical factors for the adsorption of aluminum on magnetic hydrogel beads. After identifying the significant factors using PBD, response surface methodology (RSM) was employed to optimize the parameters for aluminum adsorption.

The RSM is a powerful statistical approach that investigates and optimizes the effects of multiple independent variables on a specific response. It can be applied to investigate the various factors affecting metal adsorption efficiency and optimize the process conditions to obtain maximum adsorption.^29−31^ The RSM also provides insights into interactions among variables and aids in determining the most influential factors, ultimately reducing experimental runs, constructing mathematical models, and predicting optimal process conditions.^32^

Box–Behnken design (BBD) and central composite design (CCD) are two widely used RSM designs in experimental studies. BBD is a three-level design, and compared to full-factorial designs, it requires fewer experimental runs, making it highly applicable to studies with limited resources or a large number of factors. In contrast, the CCD is a factorial design with additional center and axial points, providing comprehensive coverage of the response surface. These designs enable researchers to optimize process parameters, identify influencing factors, and gain insights into the relationships between variables, thereby contributing to improved efficiency and effectiveness in experimental studies.^33^

This study, with the objective of reducing aluminum contamination in water sources, was carried out using an adsorbent designed with low-cost, abundant, and ecofriendly biopolymers and aimed to minimize the risks to human health by using reusable hydrogel beads. Nonmagnetic alginate beads were used earlier for the removal of toxic divalent cations, but they have not been employed for the removal and detection of Al(III). As stated in the literature, Al(III) can cause serious health disorders such as Alzheimer’s disease, dementia, anemia, and bone disorders. Therefore, it is important to develop new methods for the preconcentration, determination, and removal of Al(III) using novel adsorbents. The alginate–gelatin magnetic beads produced in this study were fully characterized by using chemical, physical, and mechanical methods. The magnetic beads demonstrated the effective, rapid, and facile adsorption of aluminum in aqueous solutions. Following the adsorption process, aluminum was efficiently recovered from the beads using HNO_3_, and the reusability of the beads was investigated. All of the adsorption and desorption studies were statistically optimized, and aluminum adsorption was investigated through thermodynamic, isotherm, and kinetic studies. The collective findings from these investigations highlight the potential of the magnetic core–shell architecture of hydrogel beads for various environmental applications that require the efficient and facile adsorption and recovery of pollutants.

## 2 Materials and Methods

### 2.1 Reagents and Apparatus

FeCl_2_·4H_2_O, FeCl_3_·6H_2_O, Al(NO_3_)_3_·9H_2_O, HCl, HNO_3_, NaOH, CaCl_2_, alginic acid sodium salt, porcine skin gelatin type A, GA (%25), absolute ethanol, and morin hydrate were purchased from Sigma-Aldrich (USA).

A solution of 1000 mg L^–1^ Al(III) was prepared by dissolving an appropriate amount of Al(NO_3_)_3_·9H_2_O in ultrapure water (UPW). A morin solution (100 mg L^–1^) was prepared by dissolving the appropriate amount of morin hydrate in absolute ethanol.

Weight measurements were performed using an AV264 balance (Ohaus, USA). A pH meter model HI-5521 (Hanna Instruments, USA) was used to measure the pH of the solutions. All of the experiments were performed in a shaking incubator (Wisd Laboratory Instruments, Germany). A freeze-dryer model 1.2 D Alpha Plus was used to prepare cryogel beads (Christ, Germany). Fourier transform infrared spectra were recorded using a Spectrum Two spectrometer (PerkinElmer, USA) in the range 4000–400 cm^–1^. X-ray photoelectron spectroscopy (XPS) measurements were performed by using a K-Alpha X-ray photoelectron spectrometer (Thermo Fisher Scientific, UK). Atomic force microscopy (AFM) measurements were performed with a Dimension Edge (Bruker, USA) in the tapping mode and an OTESPA-R3 (Bruker, USA) silicon probe in air at 25 °C. An Apreo S model scanning electron microscopy (SEM) (Thermo Fisher Scientific, USA) was used to determine the morphologies of both magnetite nanoparticles and magnetic beads. The SEM instrument, equipped with an Elect Super detector (USA) with a surface area of 70 mm^2^ and a Peltier cooling system, was used for energy-dispersive X-ray spectroscopy (EDX) analysis with a resolution of 125 eV. All samples were coated with gold/palladium (60/40) using an ACE 600 sputter coater (Leica, Germany) prior to SEM analysis. Before conducting the SEM analysis, a CPD 300 critical point dryer was used to dry the bead samples (Leica, USA). The magnetization properties were investigated by using a Lakeshore 7407 vibrating sample magnetometer (VSM, Lakeshore, USA). Zetasizer NanoZS (Malvern Instruments, UK) was used to investigate the size distribution and zeta potential of the magnetic nanoparticles. Micro-computed tomography (Micro-CT) analysis using a μCT50 device (Scanco Medical, Switzerland) was performed to determine the average pore size, porosity, and pore distribution of the magnetic beads. A uniaxial compression test was carried out on a Q800 DMA device (TA Instruments, USA) to investigate the mechanical behavior of the magnetic hydrogel beads. An energy-dispersive X-ray fluorescence (EDXRF) spectrometer (Rigaku, Japan) was used to determine the amount of Ca(II) in the solution. The emission spectra of the solutions were recorded with a Nanodrop 3300 instrument (Thermo Fisher Scientific, USA) at 488 nm as the maximum emission wavelength. Results obtained with the proposed method were compared with those of an Optima 7000 DV inductively coupled plasma optical emission spectrometer (ICP-OES) (PerkinElmer, USA). Visual Minteq software (KTH, Sweden) was used to determine the dominant aluminum species. Design Expert version 11 software (Stat-Ease, USA) was used for design of experiments (PBD, BBD, and CCD).

### 2.2 Synthesis of Magnetite Nanoparticles

Coprecipitation, one of the most widely used methods for magnetite synthesis, was used to produce the magnetite nanoparticles used in this study.^34,35^ Total 0.01 mol (1.99 g) of FeCl_2_·4H_2_O and 0.02 mol (5.41 g) of FeCl_3_·6H_2_O were dissolved in 100 mL of UPW, and the mixture was mechanically stirred. The mixture was degassed with N_2_ gas, while the temperature of the water bath to 80°–90 °C. 50 mL of 2 mol L^–1^ NaOH solution was added to the solution, resulting in black precipitates immediately. After 2 h, magnetite nanoparticles were isolated from the solution using a magnet and washed several times with UPW.

### 2.3 Synthesis of Magnetic Hydrogel and Cryogel Beads

Solution A was prepared by dissolving 0.6 g of alginic acid sodium salt in 100 mL of UPW. Solution B was prepared by dissolving 5 g of porcine skin gelatin type A and 0.75 g of CaCl_2_ in 50 mL of UPW and stirring overnight at 50 °C. Solution A was poured into a beaker and magnetically stirred at 70 °C. Magnetite nanoparticles were added to solution B at a concentration of 0.1 g L^–1^, and a syringe was filled with this solution. Magnetic hydrogel beads were formed by adding solution B drop by drop to solution A. The beads were rinsed three times with UPW. The beads were stirred overnight in a cross-linker solution consisting of 5 mL of GA in 500 mL UPW. After cross-linking, the magnetic beads were rinsed with UPW. The magnetic hydrogel beads were frozen in UPW at −80 °C and subsequently freeze-dried to obtain cryogel beads.^36^ The synthesis of this is shown in Figure S1.

### 2.4 Chemical Characterization of Magnetite Nanoparticles and Beads

#### 2.4.1 Fourier Transform Infrared Spectroscopy

All of the samples were measured using the attenuated total reflection (ATR) mode coupled with a diamond crystal. After ensuring that the sample completely covered the ATR crystal, we analyzed it using a pressure device on the instrument to ensure good contact with the crystal. The resolution was set to 2 cm^–1^, and the transmittance was selected as the output.

#### 2.4.2 XPS

To obtain XPS spectra, the samples were mounted on a sample holder and introduced into the XPS vacuum chamber. The spot size was set to 300 μm, and Al Kα radiation was used as the excitation source.

#### 2.4.3 Energy-Dispersive X-ray Spectroscopy

EDX was used to determine the chemical compositions of the materials. The samples were mounted on aluminum stubs with double-sided carbon tape and analyzed under high vacuum (<1 × 10^–3^ Pa) with an accelerating voltage of 30.0 kV and a working distance of 10 mm.

### 2.5 Physical Characterization of Magnetite Nanoparticles and Beads

#### 2.5.1 Vibrating Sample Magnetometry

The magnetic behavior of the magnetite nanoparticles and magnetic beads was investigated using VSM. The samples were analyzed in powder form in the range of −1 to 1 T at room temperature.

#### 2.5.2 Atomic Force Microscopy

AFM measurements were performed to determine the diameters of the magnetite nanoparticles. Nanoparticles were observed on the coverslip, and before sample preparation, the coverslip was cleaned with absolute ethanol. The nanoparticles were dispersed in UPW in an ultrasonic bath for 1 h. A drop of the solution was placed on a coverslip and allowed to air-dry. AFM images were acquired at a scan rate of 1.0 Hz over a scan area of 2 × 2 μm^2^ with 256 samples per scan line.

#### 2.5.3 Dynamic Light Scattering

Dynamic light scattering (DLS) was used to investigate the size distribution and zeta potential of the magnetic nanoparticles. The nanoparticles were added to UPW at a concentration of 0.1 mg mL^–1^ and dispersed by using an ultrasonic bath for 1 h. Data were obtained at 25 °C from 1 mL aliquots.

#### 2.5.4 Scanning Electron Microscopy

For the SEM analysis, the samples were mounted on aluminum stubs using double-sided carbon tape. The SEM analyses of the magnetite nanoparticles and the cross-sections of the magnetic beads were carried out under high vacuum (<1 × 10^–3^ Pa) with an accelerating voltage of 7.5 kV and spot size of 9. The outer surface of the beads was scanned under a low vacuum (50 Pa) with an accelerating voltage of 15 kV and a working distance of 49.5 mm.

#### 2.5.5 Micro-Computed Tomography

The scan parameters are set as follows: 70 kVp energy, 114 μA intensity, 10 μm voxel size, and a 300 ms integration time. Two-dimensional (2D) cross-sectional images were analyzed, and 3D models were obtained using the Evaluation Program V6.5. The porosity was calculated as follows
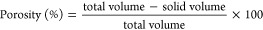
1

### 2.6 Mechanical Characterization of Beads with Dynamic Mechanical Analysis

Dynamic mechanical analysis (DMA) was used to determine the differences in the mechanical strength of the magnetic beads with and without GA cross-linking. Samples with a 3.82 ± 0.04 mm diameter and a 5.67 ± 0.8 mm thickness were placed between the compression clamps. Tests were performed at 25 °C on three replicates with a ramping force of 0.5 N/min up to 18 N.

### 2.7 Determination Procedure for Quantitative Aluminum Detection

The highly fluorescent Al(III)–morin complex was used to detect the presence of aluminum. First, the pH of the Al(III) solution was adjusted to 3.5 with HCl–NaOH. Then, 0.16 mL of 100 mg L^–1^ morin solution was added to 0.5 mL of Al(III) solution, and the mixture was diluted to 1 mL with absolute ethanol.^37,38^

### 2.8 Adsorption and Recovery Studies

For the adsorption of aluminum, 5 mL of the aluminum solution in UPW at a concentration of 1 mg L^–1^ (with pH values in the range of 4.0–9.0) was added to different amounts of the adsorbent. The mixture was shaken for 5–45 min at 25 and 45 °C. After adsorption, the magnetic hydrogel beads were separated from the solution using a magnet, and the aluminum in the solution was measured, as described in Section 2.7.

As a first step, the above adsorption procedure with optimum parameters was followed for aluminum recovery. Different concentrations of HCl, HNO_3_, and CH_3_COOH were used to quantitatively measure the recovery of aluminum. Based on these experiments, HNO_3_ at a concentration of 1 mol L^–1^ was used as the recovery agent. Thus, in the final recovery step after adsorption, the magnetic hydrogel beads were separated using a magnet, and 5 mL of 1 mol L^–1^ HNO_3_ was added to the adsorbent and shaken for 40 min to recover adsorbed aluminum.

### 2.9 Statistical Design of Experiment and Optimization Studies of Adsorption

#### 2.9.1 Plackett–Burman Design

In this study, PBD was used to determine the most effective parameters for the adsorption of aluminum on magnetic hydrogel beads. The design parameters chosen for the PBD are as follows: (i) initial pH, (ii) agitation speed, (iii) amount of adsorbent, (iv) temperature, (v) contact time, (vi) type of adsorbent, and (vii) GA cross-linking. The adsorption efficiency (%) was used as the response. The five assigned numeric (i–v) and two categoric (vi and vii) factors resulted in 12 runs per two-level PBD (Table 1). All experiments were performed in triplicate at a 95% confidence level.

**Table 1:** Optimization Parameters and Levels of PBD (Number of Numeric Factors: 5; Number of Categorical Factors: 2; and Number of Levels: 2)

factor	factor code	type	low level	high level
pH	A	numeric	2	4
agitation speed (rpm)	B	numeric	100	200
amount of adsorbent (mg)	C	numeric	100	1000
temperature (°C)	D	numeric	25	45
contact time (min)	E	numeric	2	20
type of the adsorbent	F	categoric	hydrogel	cryogel
GA cross-linking	G	categoric		+

PBD is based on a first-order model equation without any interactions among the independent factors.^39^ The obtained equation is as follows
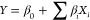
2where *Y* is the measured response (adsorption efficiency), β_0_ is the model intercept, β_*i*_ is the linear coefficient, and *X*_*i*_ is the level of each independent variable.

#### 2.9.2 RSM Using BBD

BBD is widely used to optimize complex processes in various fields owing to its simplicity, flexibility, and efficiency in determining the optimum conditions for a given process. It is an RSM that helps create a regression model for a process by fitting a quadratic model to the experimental data.^40^

Based on the results obtained by screening different parameters using PBD, the amount of adsorbent, pH, and contact time were found to be the three most significant parameters for the adsorption of aluminum. In the BB experiments, the temperature was maintained at 25 °C with an agitation speed of 150 rpm, and the GA-cross-linked hydrogel was used as the adsorbent. These parameters were kept constant for all experiments. The experimental design of BBD (Table 2) resulted in 15 runs for the three independent variables. All experiments were carried out in triplicate at a 95% confidence level.

**Table 2:** Optimization Parameters and Levels of BBD (Temperature: 25 °C; Agitation Speed: 150 rpm; and Adsorbent type: GA-Cross-Linked Hydrogel)

		levels		
factor	factor code	low	center	high
pH	A	4.0	6.5	9.0
amount of adsorbent (mg)	B	200.0	600.0	1000.0
contact time (min)	C	5.0	25.0	45.0

The experimental data were fitted to a quadratic model using a second-order polynomial as follows
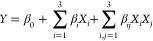
3where *Y* is the measured response (adsorption efficiency, %); β_0_ is the model intercept; β_1_, β_2_, and β_3_ are the linear regression coefficients; β_12_, β_13_, and β_23_ are the interaction coefficients; β_11_, β_22_, and β_33_ are the quadratic regression coefficients; and *X*_1_, *X*_2_, and *X*_3_ are the coded values of the significant independent variables.

### 2.10 Optimization Studies of Recovery

CCD was used to optimize the recovery of aluminum from GA-cross-linked magnetic hydrogel beads by varying the reagent concentration and recovery time. As BBD requires at least three factors,^41^ it could not be used to optimize the recovery process.

To desorb aluminum ions from the adsorbent, preliminary studies were carried out with a number of reagents, including HNO_3_, HCl, CH_3_COOH, and NaOH at 1 mol L^–1^. As shown in Table S1, the beads were dissolved in 1 mol L^–1^ HCl, and the recovery values for CH_3_COOH and NaOH were lower than 50%. Quantitative recovery was obtained using only 1 mol of L^–1^ HNO_3_. Thus, CCD was used only to optimize the concentration of HNO_3_ and the recovery time. The experimental design of the CCD, including both the factorial and axial points, is presented in Table S2. The CCD resulted in 11 runs for two independent variables. All experiments were carried out in triplicates at a 95% confidence level. The experimental data were fitted to a quadratic model by using a second-order polynomial.

### 2.11 Statistical Analysis

All quantitative data values are presented as the mean ± standard deviation of experiments performed with at least three replicates (*n* = 3). Statistical significance was determined by one-way analysis of variance (ANOVA) at a 95% confidence interval using GraphPad Prism version 8.1.0. Multiple comparisons were performed using Tukey’s post hoc method.

## 3 Results and Discussion

### 3.1 Characterization of Magnetite and Beads

#### 3.1.1 Chemical Characterization

##### 3.1.1.1 Fourier Transform Infrared Spectroscopy

**Figure 1:**
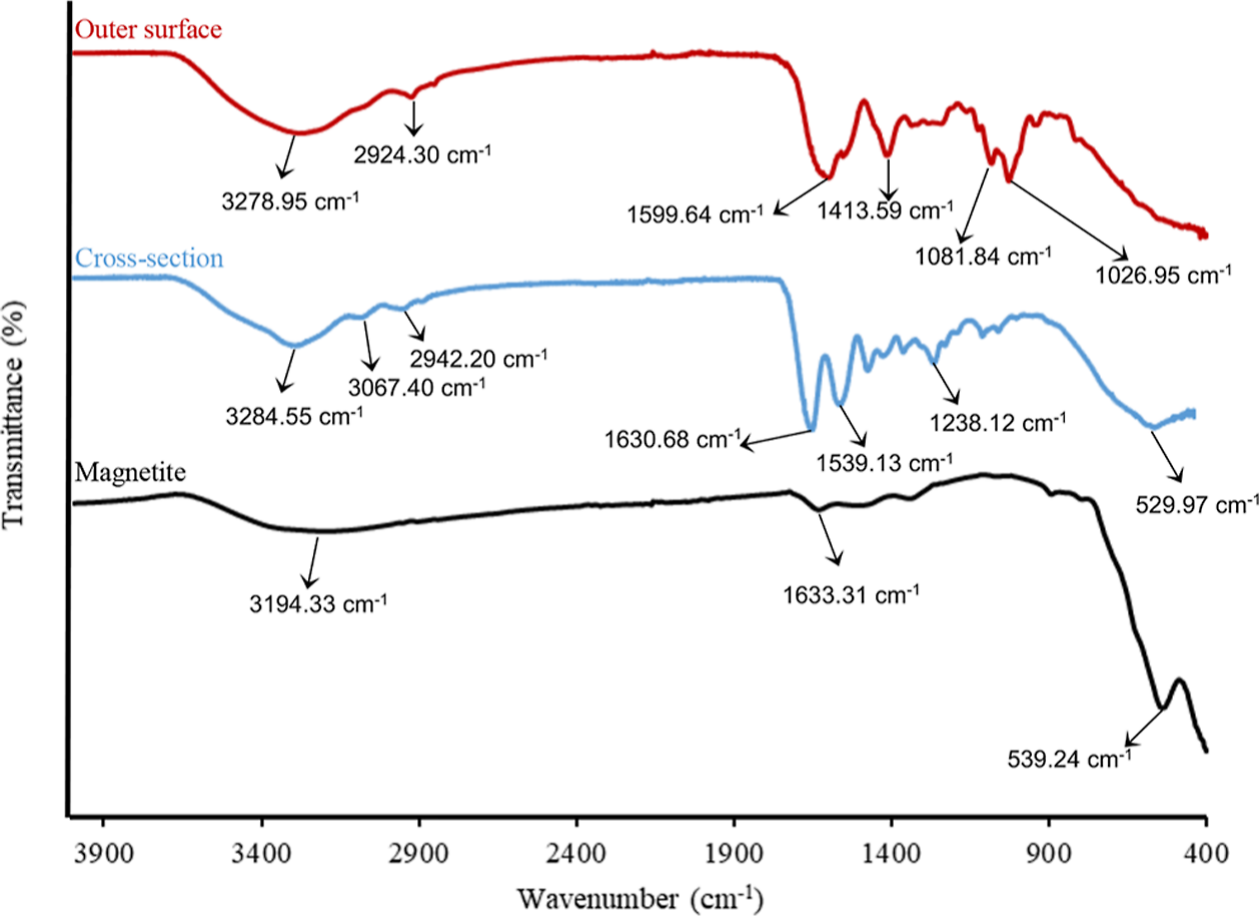
FTIR spectra of magnetite nanoparticles, outer surface, and cross-section of the magnetic beads (mode: ATR; output: transmittance; and resolution: 2 cm^–1^).

Figure 1 shows the Fourier transform infrared spectroscopy (FTIR) spectra of the magnetite nanoparticles and the surface and cross-sections of the magnetic beads.

In the FTIR spectrum of magnetite nanoparticles, only Fe–O peaks should be observed due to the chemical structure of magnetite. The peak at approximately 540 cm^–1^ is associated with the stretching of the Fe–O bond,^42−44^ indicating the successful synthesis of magnetite nanoparticles. However, upon water adsorption on the particle surface, peaks corresponding to O–H stretching and O–H bending vibrations arising from the adsorption of water molecules can be observed at 3194.33 and 1633.31 cm^–1^, respectively.^45^ Therefore, the FTIR spectrum provides evidence of the synthesis of magnetite particles with water molecules adsorbed onto their surface.

For the cross-section of the magnetic bead, the structure would consist of magnetite nanoparticles and gelatin cross-linked with glutaraldehyde. As a result, the FTIR spectrum should exhibit characteristic peaks corresponding to gelatin. Related to the cross-section of the magnetic bead, the characteristic peaks of gelatin, such as amide-A (N–H stretching), amide-B (N–H bending, – CH_2_ stretching), amide-I (C=O stretching), amide-II (N–H stretching, C–N bending), and amide-III (C–N stretching, N–H phase bending) were observed at 3284.55, 3067.40–2942.2, 1630.68, 1539.13, and 1238.12 cm^–1^, respectively.^46−49^ Additionally, it is expected that the peaks related to magnetite particles will also be present in the cross-sectional structure. The presence of magnetite particles within the structure is confirmed by the appearance of the Fe–O band of the magnetite nanoparticles, which was slightly shifted to 529.97 cm^–1^.

For the outer surface of the magnetic bead, the structure is expected to be composed of an alginate structure. Furthermore, with the successful coating of magnetite particles, the detection of peaks related to magnetite is not expected. The characteristic peaks of alginate such as –OH stretching at 3278.95 cm^–1^, C–H stretching at 2924.30 cm^–1^, asymmetric stretching of carboxyl at 1599.64 cm^–1^, symmetric stretching of carboxyl (COO–) at 1413.59 cm^–1^, and C–O–C stretching at 1081.84 and 1026.95 cm^–1^ are observed.^27,50−52^ These observed peaks confirm the successful synthesis of the expected structure for the outer surface of the beads. As anticipated, no peak corresponding to the Fe–O band was observed, further confirming successful encapsulation.

##### 3.1.1.2 X-ray Photoelectron Spectroscopy

**Figure 2:**
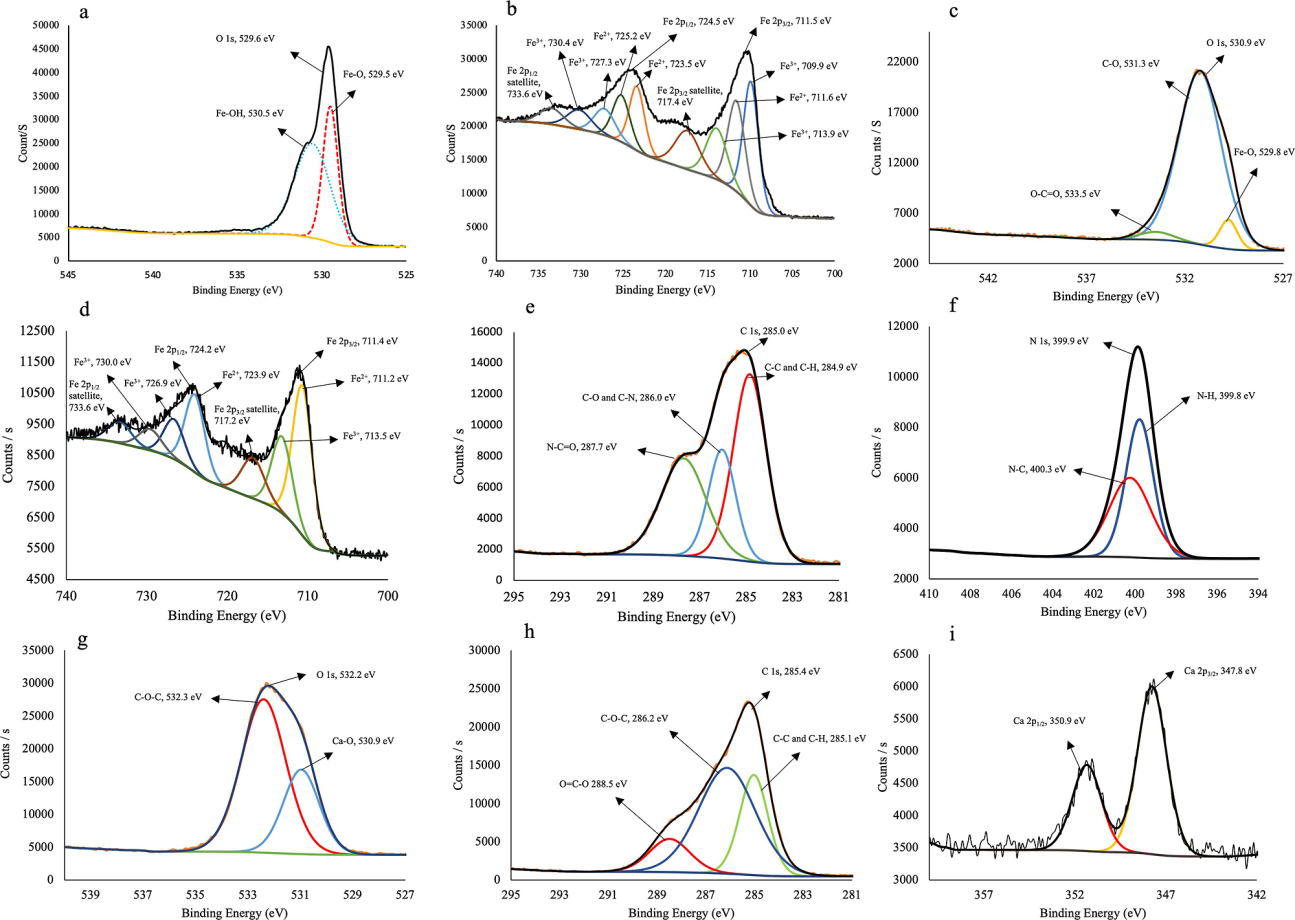
XPS spectra of (a) O 1s scan and (b) Fe 2p scan of magnetite nanoparticles; (c) O 1s scan, (d) Fe 2p scan, (e) C 1s scan, and (f) N 1s scan of cross-section of the magnetic beads; and (g) O 1s scan, (h) C 1s scan, and (i) Ca 2p scan of the outer surface of the magnetic beads (spot size: 300 μm, excitation source: Al Kα radiation).

The magnetite nanoparticles and the cross-section and outer surface of the magnetic beads were characterized using XPS. As shown in Figure 2a, for the XPS O 1s scan, the oxygen was deconvoluted into two peaks. The O 1s peak at 529.5 eV can be assigned to oxygen in Fe–O,^53^ and the peak at 530.5 eV can be related to the –OH group because of the presence of Fe–OH on the surface of the magnetite nanoparticles.^54,55^

The Fe 2p_3/2_ and Fe 2p_1/2_ peaks of the magnetite nanoparticles were observed at binding energies of 711.5 and 724.5 eV, respectively. The deconvoluted peaks of Fe^2+^ and Fe^3+^ are shown in Figure 2b. The peaks at 711.6, 723.5, and 725.2 eV correspond to the Fe–O bond of the Fe^2+^ ion, whereas the peaks at 709.9, 713.9, and 727.3 eV can be assigned to the Fe–O bond of the Fe^3+^ ion. Furthermore, the small satellite peaks at 719.2 and 733.6 eV can be attributed to Fe^3+^.^56,57^ The Fe 2p XPS spectrum provided evidence for the presence of both Fe^2+^ and Fe^3+^ states, confirming that the nanoparticles are indeed magnetite.^58^

The XPS analysis of the cross-section of the magnetic bead is shown in Figure 2c–f. The XPS spectrum revealed the presence of Fe, O, C, and N, which are present in magnetite nanoparticles, gelatin, and GA, as confirmed by FTIR analysis. O 1s (530.9 eV) was deconvoluted into three peaks at 529.8, 531.3, and 533.5 eV (Figure 2c). The peak at 529.8 eV was caused by Fe–O,^53^ the peak at 531.3 eV by C–O, and the peak at 533.5 eV is because of the O–C=O group.^59^ Fe 2p peaks are observed as Fe 2p_3/2_ (711.4 eV) and Fe 2p_1/2_ (724.2 eV), originating from the magnetite nanoparticles in the structure. The deconvoluted peaks at 711.2, 713.5, 717.2, 723.9, 724.2, 726.9, 730, and 733.6 eV are shown in Figure 2d.^56,57^ The C 1s peak can be seen at 285.0 eV and is deconvoluted into three peaks (Figure 2e). The peak at 284.9 eV is attributed to C–H/C–C, the peak at 286.0 eV to C–O/C–N, and the peak at 287.7 eV is caused by N–C=O.^60−63^ The N 1s nitrogen peak caused by the presence of gelatin is observed at 399.9 eV and is deconvoluted into two peaks centered at 399.8 and 400.3 eV (Figure 2f). The peak at 399.8 eV is attributed to the N–H bond, and the peak at 400.3 eV corresponds to the N–C bond.^60,64^

The XPS spectra of the outer surface of the magnetic beads revealed the presence of C, Ca, and O in alginate cross-linked with calcium, as shown in Figure 2 g–i. C 1s, centered at 285.4 eV, is deconvoluted into three peaks at 285.1, 286.2, and 288.5 eV, which are related to C– C/C–H, C–O–C, and O–C=O, respectively, and arise from the alginate structure (Figure 2g).^53,65^ The O 1s peak (532.3 eV) was deconvoluted into two peaks at 530.9 and 532.3 eV, which are caused by Ca–O and C–O–C, respectively (Figure 2h).^66^ Ca 2p_1/2_ and Ca 2p_3/2_ with binding energies of 350.9 and 347.8 eV, respectively (Figure 2i), indicate the existence of Ca(II) in the adsorbent. The presence of two peaks is indicative of two forms of calcium: calcium ions and associated calcium, which is consistent with the literature.^67,68^

##### 3.1.1.3 Energy-Dispersive X-ray Spectroscopy

The EDX spectra of the magnetite nanoparticles are shown in Figure S2, and their analysis (Figure S2a) shows that the structure consists of only Fe and –O, thus confirming the successful synthesis. The EDX analysis of the magnetic bead cross-section (Figure S2b) reveals that the structure contains Fe and O, as well as C and N. However, as expected, the outer surface of the beads was composed of Ca, O, and C (Figure S2c). As EDX is a surface characterization technique, the Fe peak was not observed on the surface of the synthesized beads, which confirms the successful encapsulation of the magnetite nanoparticles, as observed in both FTIR and XPS analyses.

EDX mapping analysis provides information about the elemental composition and distribution within the sample. Mapping technique helps to visualize variations in elemental concentrations and understand the spatial arrangement of elements within the sample. Different colors are often used to represent specific elements or elemental compositions on the sample’s surface.

**Figure 3:**
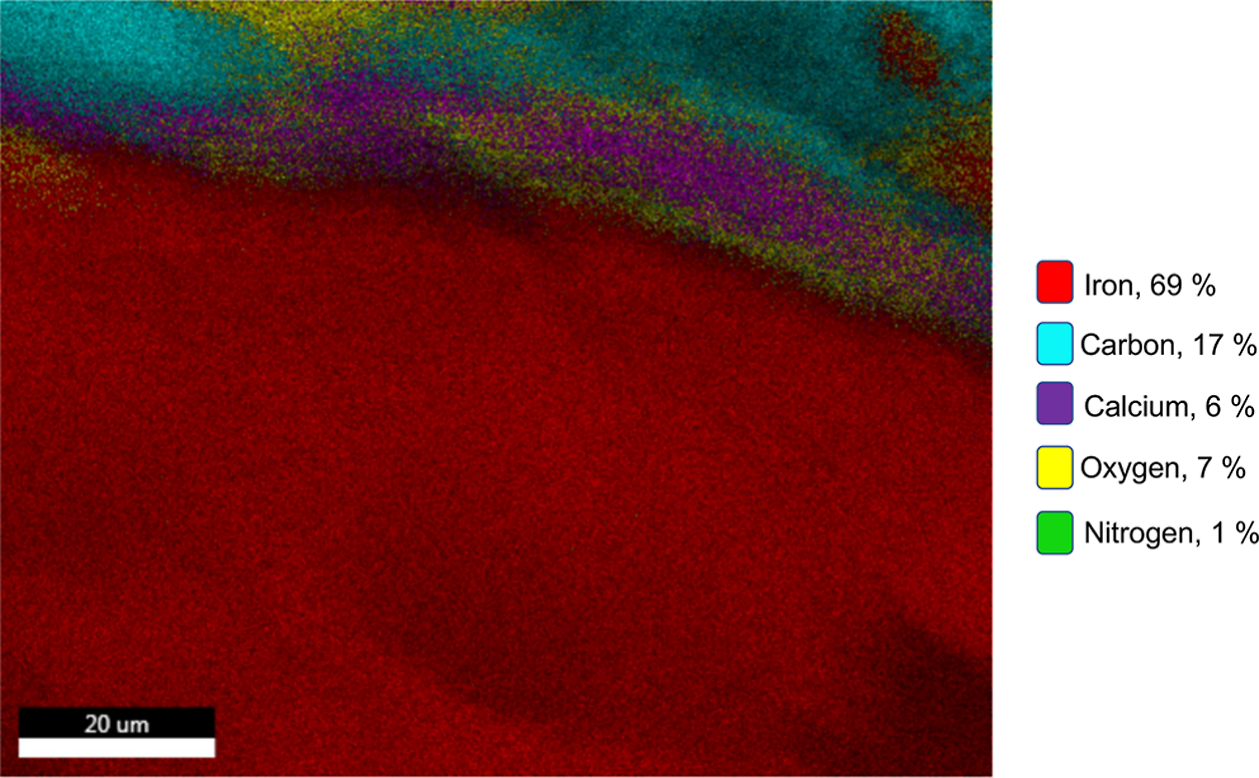
EDX mapping analysis of the cross-section of magnetic beads (accelerating voltage: 30.0 kV; working distance: 10 mm; magnification: 3500×; and resolution: 130.7 eV).

Figure 3 illustrates the EDX mapping analysis of the cross-section of magnetic beads. The mapping analysis was conducted on this specific region (Figure S3a) due to the potential for identifying structural differences. The outer surface was anticipated to consist of alginate cross-linked with calcium. Therefore, the outer surface should primarily contain Ca, O, and C. As evident from the EDX mapping analysis, calcium is detected solely on the outer surface of the bead. Thus, it can be concluded that calcium cross-linking occurs only on the outer surfaces of the beads. Besides the other components used, gelatin is the only component that contains the nitrogen element. Therefore, the presence of nitrogen on the interface proves the presence of gelatin. Gelatin, which is cross-linked with GA, not only enhances the stability of magnetite nanoparticles in the structure but also prevents the leakage of the nanoparticles. Magnetite is the sole component that contains the iron (Fe) element, and as can be seen from the mapping analysis, Fe is exclusively detected in the cross-section of the bead, with no Fe detected on the outer surface. This result provides evidence of the successful encapsulation of the magnetite particles within the organic structure. Furthermore, as depicted in Figure S3a, the SEM image clearly displays the structural differences between the inner and outer surfaces of the beads. Additionally, Figure S3b presents the EDX spectrum of the relevant cross-section, encompassing all of the elements observed in the mapping analysis.

#### 3.1.2 Physical Characterization

##### 3.1.2.1 VSM

The magnetic behavior of the bare magnetite nanoparticles and magnetic beads was investigated by using the hysteresis curve obtained from VSM analysis. The saturation magnetization (M_S_) values obtained are 27.3 and 8.73 emu g^–1^ for bare magnetite nanoparticles and GA cross-linked magnetic beads, respectively, which are appropriate for the magnetic separation (see Figure S4).^69^ The nonmagnetic shell coating on the magnetite nanoparticles causes a significant decrease in the M_S_ value, which confirms the successful coating and encapsulation of magnetic nanoparticles in the GA cross-linked magnetic beads.^70^

##### 3.1.2.2 Atomic Force Microscopy

**Figure 4:**
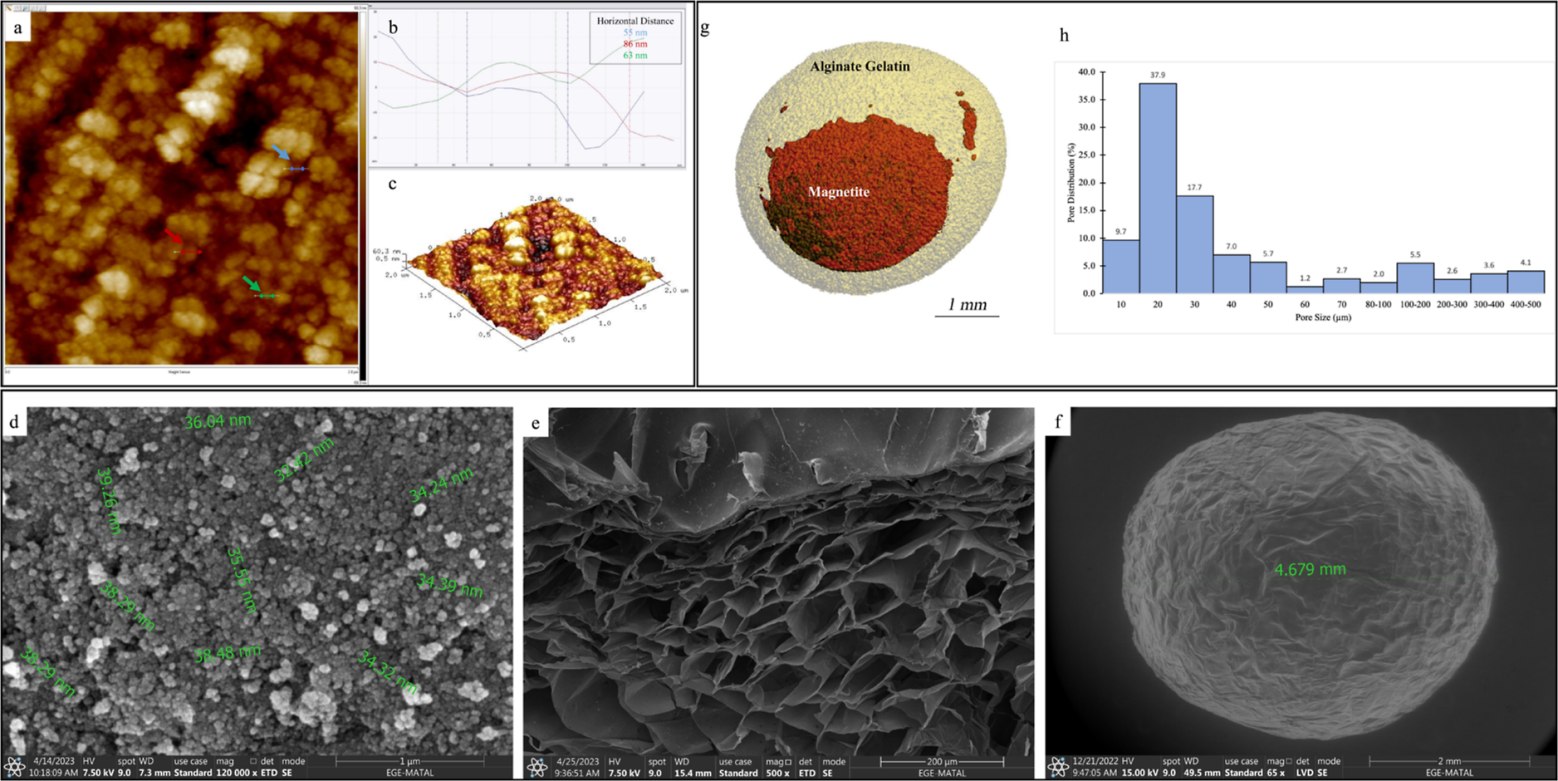
(a) 2D image of AFM scan of magnetite nanoparticles, (b) measurement of diameters of nanoparticles, (c) 3D topography image of the nanoparticles, d) SEM image of magnetite nanoparticles, (e) SEM image of cross-section of the bead, (f) SEM image of the outer surface of the magnetic beads, (g) 3D model of the GA cross-linked magnetic bead created with micro-CT analysis, and (h) pore distribution histogram of beads.

AFM measurements were performed on dried magnetite nanoparticles deposited on glass surfaces. All images were acquired over a 2 × 2 μm^2^ scan area, as shown in Figure 4a–c and analyzed using NanoScope Analysis 1.5 Software. The mean diameter of the nanoparticles, as determined from the 2D image, was 55–86 nm. The AFM results were consistent with the SEM measurements, indicating that the particle size was less than 100 nm.

##### 3.1.2.3 Dynamic Light Scattering

From the DLS results, the hydrodynamic diameter of the magnetite nanoparticles was found to be 203.39 ± 0.49 nm (PDI 0.272), and the zeta potential of the particles was determined to be −36.5 ± 7.22 mV. Figure S5 shows the particle size distribution. The hydrodynamic diameter can vary based on the properties of the liquid medium in which the nanoparticles are dispersed and is influenced by the molecules or ions that are attached to its surface.^71^ Therefore, this result does not always correspond to the physical size of the nanoparticle. Thus, it is acceptable for the hydrodynamic diameter obtained by DLS to be larger than the physical diameter of the nanoparticles measured by AFM and SEM. A PDI value of less than 0.5 indicates that the nanoparticles are close to monodisperse, i.e., they have similar sizes.^72^ Additionally, a high zeta potential indicates a stable dispersion of the nanoparticles in UPW.

##### 3.1.2.4 Scanning Electron Microscopy

The magnetite nanoparticles had a spherical shape with an average size of 36.1 ± 2.3 nm (*n* = 10), as shown in Figure 4d. The porous structure of the bead can be seen in the cross-sectional image (Figure 4e) and its spherical shape, with a diameter of approximately 4.7 mm, are shown in Figure 4f.

##### 3.1.2.5 Micro-Computed Tomography

Micro-CT scanning was performed only on the magnetic cryogel beads (*n* = 3) because the hydrogels could not be accurately scanned. Magnetic cryogel beads were placed in standard sample holders and scanned, and 2D images were reconstructed. A 3D model was generated using the Evaluation Program (Figure 4g), showing the localization of magnetite nanoparticles (red) in the beads (light yellow). The histogram in Figure 4h shows that the pore sizes in the beads are in the range 10–500 μm. The highest frequencies were observed for the 20 (37.9%) and 30 μm (17.7%) pore sizes. The micro-CT evaluation revealed that the mean pore diameter and porosity of the beads were 66.9 μm and 35.56%, respectively.

#### 3.1.3 Mechanical Characterization of the Beads

A uniaxial compression test was performed to determine the mechanical properties of the magnetic beads with and without GA cross-linking. The Young’s moduli of the magnetic beads calculated from the slope of the linear part of the stress–strain curve (Figure S6) were 41.2 and 132.3 kPa, respectively. On the magnetic beads without GA cross-linking, a compression test was performed for 11.084 min, with a static force of 5.52 N applied at the end. On GA-cross-linked magnetic beads, in contrast, the test was carried out for a longer period of 16.76 min with a higher applied static force of 8.36 N. The higher values of Young’s modulus and static force observed for the GA-cross-linked magnetic beads indicate that they have greater strength. This strength is particularly important in adsorption and desorption as it affects hydrogel degradation and reusability rates.^73^ Cross-linking during adsorbent synthesis enhances its structural integrity, providing robustness and preventing any potential decomposition during the adsorption. The improved stability ensures that the adsorbent maintains its sorption efficiency and performance over multiple cycles. In this study, we chose GA-cross-linked magnetic beads to prevent the release of magnetite nanoparticles from the beads and to achieve higher reusability rates.

### 3.2 Screening of Significant Variables Using PBD

PBD was carried out for five numerical parameters (pH, agitation speed, amount of adsorbent, temperature, and contact time) and two categorical factors (type of adsorbent and GA cross-linking). The influence of these parameters on the adsorption of aluminum was statistically analyzed using PBD. The experimental design for the adsorption process is presented in Table 3.

**Table 3:** Experimental Design According to Two-Level PBD and Experimental and Predicted Values of Adsorption Efficiency (%) (Number of Numerical Factors: 5; Number of Categorical Factors: 2; and Number of Runs: 12)

								adsorption efficiency	
run	pH	speed (rpm)	amount (mg)	temp. (°C)	time (min)	type	GA	experimental (%)	predicted (%)
1	2	200	1000	25	20	hydrogel		30.12	33.09
2	4	200	1000	25	2	cryogel		45.09	40.42
3	2	100	1000	25	20	hydrogel	+	22.40	23.79
4	2	200	1000	45	2	cryogel	+	98.23	93.24
5	4	100	1000	45	20	cryogel	+	8.43	4.70
6	4	100	1000	45	2	hydrogel		44.18	48.85
7	2	100	100	45	2	hydrogel		52.70	51.31
8	4	200	100	45	20	hydrogel	+	18.21	21.62
9	2	100	100	25	2	cryogel	+	64.15	67.88
10	4	100	100	25	20	cryogel		51.30	54.26
11	4	200	100	25	2	hydrogel	+	5.19	2.54
12	2	200	100	45	20	cryogel		59.00	57.29

**Figure 5:**
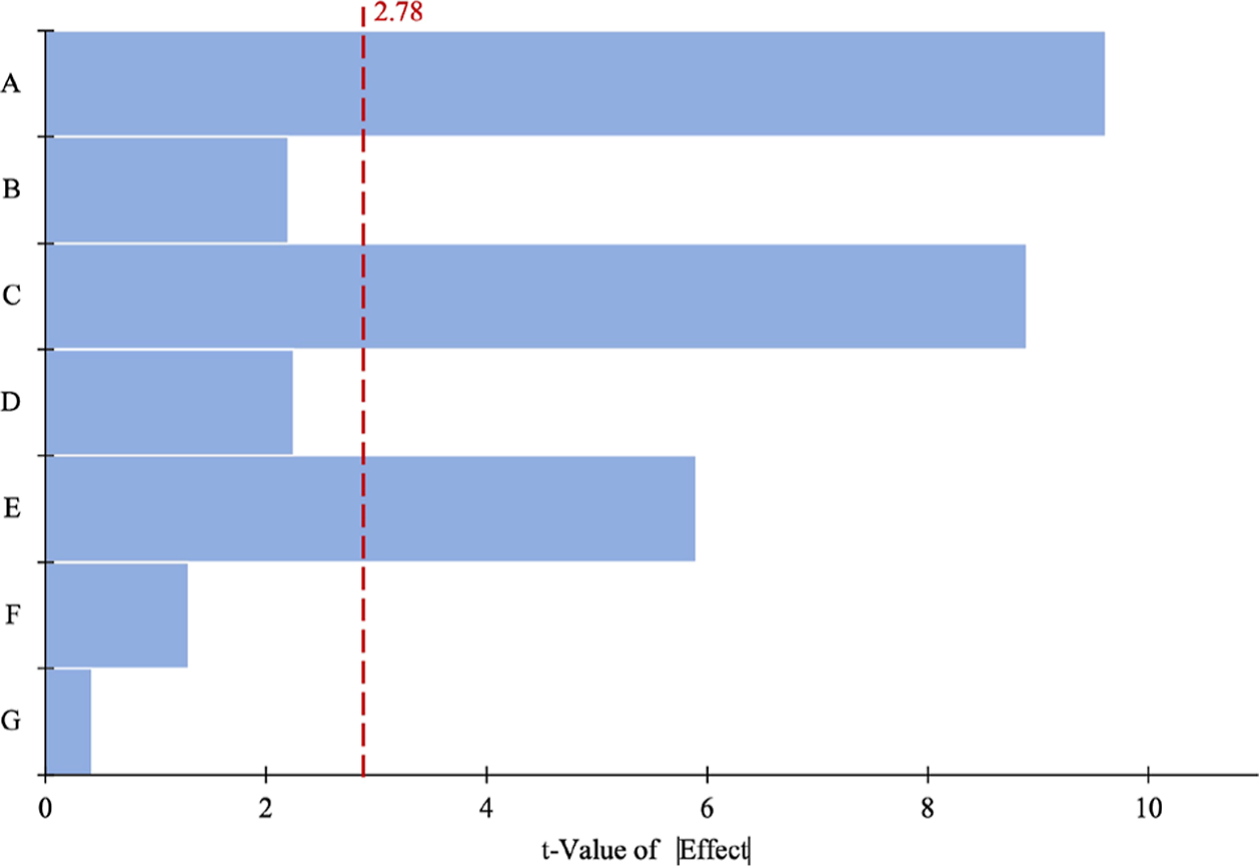
Pareto chart of the design parameters, indicating the significance factors.

The *p*-values of the amount of adsorbent, pH, and contact time were found to be lower than 0.005; therefore, these factors were identified as significant parameters and chosen for the next level of optimization by the BBD of the RSM (Table 4). The significant contributions of parameters A–E are also presented in Figure 5 using a Pareto chart, which is the most effective way to show PBD results. The importance of the effects and the magnitude are also presented in Figure 5 using a Pareto chart, which is the most effective way to display PBD results. The t-Value limit (2.78), a specific threshold level, can be used to indicate which factors are considered significant at the 0.05 level on the chart. In this model, it has been determined that the parameters A, C, and E are statistically significant, as they surpass the reference value of 2.78.

**Table 4:** ANOVA of PBD for the Aluminum Adsorption Process^a^

source	sum of squares	degree of freedom	mean square	*F*-value	*p*-value
model	7588.38	7	1084.05	31.1000	0.0025
pH	3219.65	1	3219.65	92.3600	0.0007
speed	161.04	1	161.04	4.6200	0.0980
amount	2776.74	1	2776.74	79.6600	0.0009
temperature	166.36	1	166.36	4.7700	0.0943
contact time	1208.41	1	1208.41	34.6700	0.0042
type	52.50	1	52.50	1.5100	0.2870
GA	3.67	1	3.67	0.1054	0.7617

a *R*^2^ = 0.9820; adjusted *R*^2^ = 0.9504; and predicted *R*^2^ = 0.8376.

The final equation in terms of the coded factors is as follows
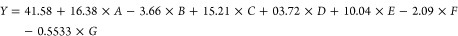
4where *Y* is the response (adsorption efficiency, %) and *A*, *B*, *C*, *D*, *E*, *F*, and *G* are the coefficients of pH, agitation speed, amount of adsorbent, temperature, contact time, type of adsorbent, and GA cross-linking, respectively.

### 3.3 Statistical Design of the Experiment and Optimization of Adsorption Variables Using BBD

RSM using BBD was carried out to obtain the optimum values of the most important independent parameters with a minimum number of runs.^74^ The effects of the pH, amount of adsorbent, and contact time were studied. Fifteen experiments were performed, with three replicates and three center points. The experimental and predicted values of the adsorption efficiency of BBD are listed in Table 5.

**Table 5:** Experimental Design According to BBD and Experimental and Predicted Values of Adsorption Efficiency (%) (Number of Independent Parameters: 3; Number of Runs: 15; and Number of Center Points: 3)

				adsorption efficiency	
run	pH	amount (mg)	time (min)	experimental (%)	predicted (%)
1	4.0	200	25	80.0	77.75
2	9.0	1000	25	26.0	28.25
3	9.0	600	45	29.0	30.33
4	6.5	600	25	84.0	84.07
5	9.0	600	5	1.0	0.00
6	4.0	600	45	96.0	100.68
7	6.5	200	45	74.0	71.58
8	6.5	600	25	80.0	84.07
9	6.5	1000	5	48.0	50.43
10	4.0	1000	25	95.4	94.30
11	6.5	600	25	88.2	84.07
12	6.5	1000	45	92.0	88.43
13	9.0	200	25	10.0	11.10
14	6.5	200	5	30.0	33.58
15	4.0	600	5	60.0	58.68

The ANOVA results for the quadratic model using a second-order polynomial with an RSM using BBD are presented in Table 6. The model *F*-value is 63.53, and its *p*-value is 0.0001, signifying that the model is statistically significant.^75^ As per Mahfud and Asori, a regression model with a correlation coefficient (*R*^2^) greater than 0.90, a maximum difference of 0.2 between the *R*^2^ and adjusted *R*^2^, and a nonsignificant lack of fit indicates a strong correlation and a well-fitted model.^76^ The *R*^2^ value for this model was determined to be 0.9913, with an adjusted *R*^2^ of 0.9757, signifying an excellent match between the experimental and adjusted values. Additionally, the predicted *R*^2^ value was estimated to be 0.8920, further demonstrating a high level of accuracy.

**Table 6:** ANOVA for RSM Using BBD for Aluminum Adsorption^a^

source	sum of squares	degree of freedom	mean square	*F*-value	*p*-value	
model	14948.95	9	1660.99	63.5300	0.0001	significant
A	8804.65	1	8804.65	336.7800	<0.0001	
B	567.84	1	567.84	21.7200	0.0055	
C	2888.00	1	2888.00	110.4700	0.0001	
AB	0.09	1	0.09	0.0034	0.9555	
AC	16.00	1	16.00	0.6120	0.4694	
BC	0.00	1	0.00	0.0000	1.0000	
A^2^	1929.24	1	1929.24	73.7900	0.0004	
B^2^	257.95	1	257.95	9.8700	0.0256	
C^2^	798.78	1	798.78	30.5500	0.0027	
residual	130.72	5	26.14			
lack of fit	97.09	3	32.36	1.9200	0.3599	not significant
pure error	33.63	2	16.81			
total	15079.67	14				

a *R*^2^ = 0.9913; adjusted *R*^2^ = 0.9757; and predicted *R*^2^ = 0.8920.

The final equation in terms of coded factors is as follows
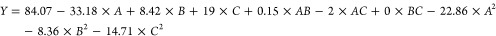
5where *Y* is the response (adsorption efficiency,%), *A* is the coefficient of pH, *B* is the coefficient of the amount of adsorbent, and *C* is the coefficient of contact time.

**Figure 6:**
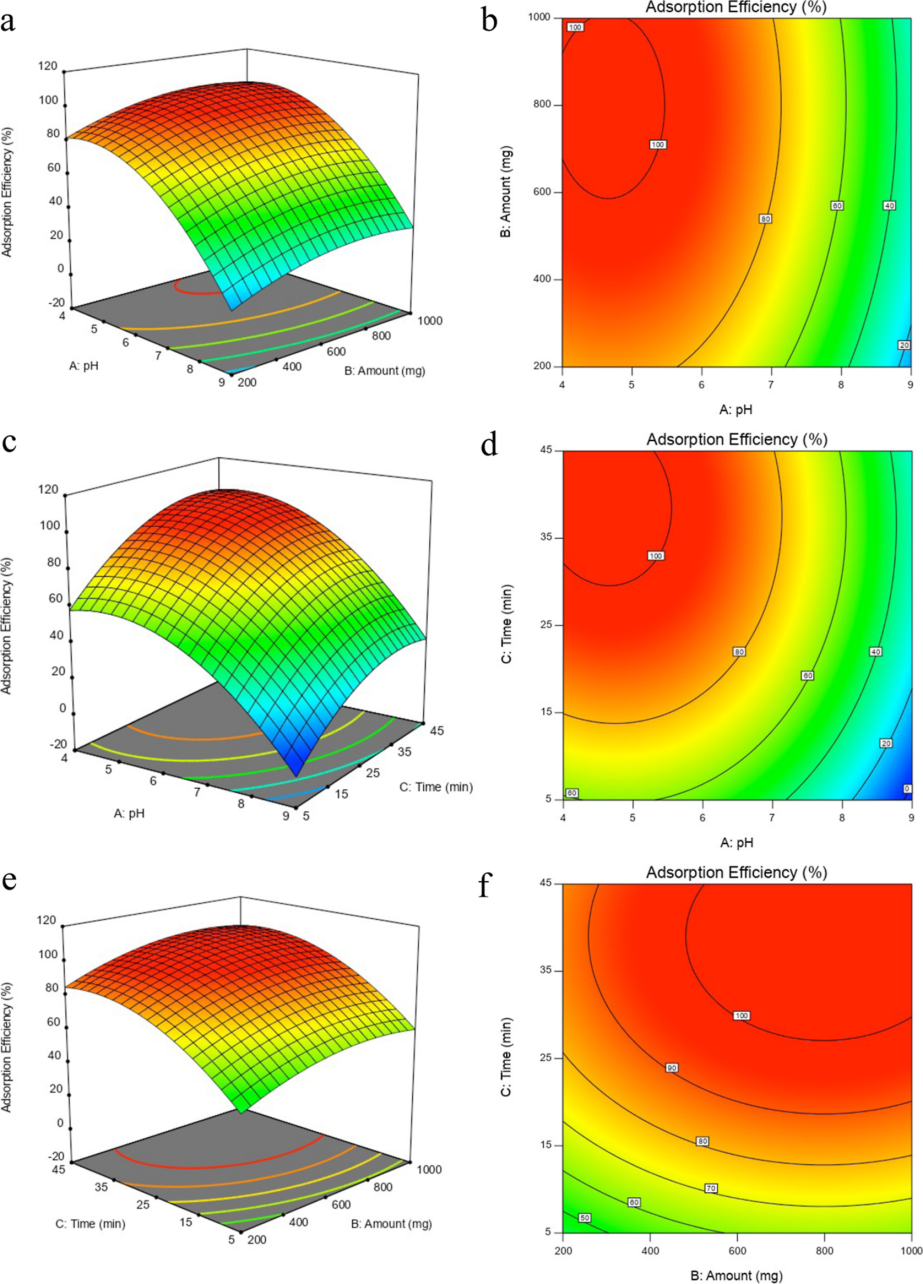
3D response surface plots of the adsorption efficiency of aluminum as a function of two independent variables: (a) pH and amount, (c) pH and time, and (e) amount and time. The contour plots of the adsorption efficiency of aluminum as a function of two independent variables: (b) pH and amount, (d) pH and time, and (f) amount and time (pH: 4–9, time: 5–45 min, and amount: 200–1000 mg).

The experimental plots were generated using eq 5. One variable was maintained constant, and the other two variables varied within the experimental range. An examination of the ANOVA results showed that the pH had the greatest impact among the three variables. The time and amount also had a significant effect on the adsorption efficiency. Figure 6a,b shows that the adsorption efficiency increased with increasing amounts of adsorbent. The response remained unchanged up to a pH of ∼5.5; however, higher values caused a dramatic decrease. A similar pattern can be seen in Figure 6c,d for pH vs contact time. When the pH is maintained constant, an increase in time positively affects the efficiency. Conversely, when the time was kept constant, a decrease in the pH significantly enhanced the efficiency. The combined effects of the adsorbent amount and contact time are shown in Figure 6e,f. As per the obtained model equation, maximizing the adsorption efficiency minimizes the amount of adsorbent and contact time; the optimized adsorption variables are a pH of 4.5, a contact time of 30 min, and an adsorbent amount of 600 mg.

The three selected confirmatory experiments generated by the model were performed in triplicate, and the fitted model was validated and confirmed. As shown in Table S3, the experimental values are close to the predicted response values.

The second-order polynomial model shows that the maximum adsorption efficiency of aluminum on GA-cross-linked hydrogel beads occurs under the following optimum conditions: pH 4.5, adsorbent amount 600 mg, and contact time 30 min at 25 °C and 150 rpm agitation speed.

Previous studies have shown that the ion-exchange mechanism is responsible for the adsorption of various divalent metal ions on calcium–alginate beads.^77−79^ In addition, trivalent ions such as La(III), Cr(III), and Nd(III) in the solution are also exchanged with Ca(II) ions in the structure.^80−82^

Visual Minteq software was used to determine the dominant species of aluminum at an optimum pH value of 4.5. At pH 4.5, aluminum was present as Al^3+^ (75.5%), AlOH^2+^ (22.1%), and Al(OH)_2_^+^ (3.4%). After the adsorption of 5 mL of 1 mg/L Al(III), the amount of Ca(II) in the solution was determined (with an EDXRF spectrometer) to be 0.204 μmol. The amount of Al(III) adsorbed by the adsorbent was recovered using fluorimetry and determined to be 0.185 μmol. Similarly, for the adsorption of 5 mL of 2 mg/L Al(III), the amounts of released Ca(II) and adsorbed Al(III) were 0.346 and 0.370 μmol, respectively, confirming that ion exchange is responsible for the adsorption process. With an increase in pH, the dominant aluminum species become negatively charged, hindering the ion-exchange mechanism. Additionally, the negatively charged alginate and aluminum species repelled each other electrostatically, resulting in a decrease in the adsorption efficiency.

### 3.4 Adsorption Kinetics

Pseudo-first-order, pseudo-second-order, and intraparticle diffusion models were used to determine the mechanism and kinetic parameters of aluminum adsorption. Total 5 mL of 20 mg/L aluminum solution (pH = 4.5) was shaken with 100 mg of the adsorbent for different periods of time, varying from 5 to 1440 min at 25 °C. The parameters of the models were calculated using the equations provided in the Supporting Information.

As shown in Table S4, the pseudo-second-order model fits the adsorption process for the highest value of *R*^2^, indicating that the adsorption rate is controlled by the chemisorption step which involves electron sharing or electron transfer between the adsorbent and the adsorbate.^83−86^

### 3.5 Adsorption Isotherms

The relationship between the adsorbate and adsorption was investigated by using Langmuir, Freundlich, and Dubinin–Radushkevich (D–R) isotherm models. Experiments were carried out by shaking 200 mg of beads with various initial concentrations (1–50 mg L^–1^) of 5 mL Al(III) solution at pH = 4.5 for 24 h. Details of the equations are provided in the Supporting Information, and the results are shown in Table S5.

As shown in Table S5, the Langmuir isotherm can be used to explain the interaction of aluminum with the magnetic beads as its *R*^2^ (0.9973) is the closest to unity. The binding sites on the adsorbent are independently occupied by the adsorbate molecules.^87^ Also, the surface of the beads is homogeneous, and monolayer adsorption occurs on the surface.^88,89^ The nature adsorption pattern considered as favorable because of the *R*_L_ value which is between 0 and 1.^90,91^

The adsorption energy was estimated from the D–R isotherm parameters. As the value of adsorption energy *E* (4.23 kJ mol^–1^) is lower than 8 kJ mol^–1^, the adsorption process is identified to be physical adsorption.^92,93^

The adsorption capacity obtained from the Langmuir isotherm model was 5.25 mg g^–1^. The adsorption capacity was also experimentally determined by shaking 200 mg of the GA cross-linked magnetic bead adsorbent with 5 mL of 100 mg L^–1^ Al(III) (pH = 4.5) for 24 h, and the value obtained was 4.15 ± 0.10 mg g^–1^ (*n* = 3). Magnetic beads without GA cross-linking were also investigated, and their adsorption capacity was determined to be 4.15 ± 0.06 mg g^–1^ (*n* = 3). As shown by the PBD, the cross-linking of GA was not an effective parameter for the adsorption process. This was confirmed by the absence of any significant difference in the Al(III) adsorption capacity.

In summary, aluminum adsorption on beads is a monolayer and physical adsorption on a homogeneous surface.

### 3.6 Thermodynamic Studies

Thermodynamic analyses were performed at three different temperatures (298, 308, and 318 K). The thermodynamic parameters for aluminum adsorption were Gibb’s free energy change (Δ*G*°), enthalpy change (Δ*H*°), and entropy change (Δ*S*°), calculated using the equations given in the Supporting Information.

As shown in Table S6, positive Δ*H*° indicates endothermic behavior of the process.^94^ Negative Δ*G*° values show the spontaneous nature of the adsorption process.^95,96^ Positive Δ*S*° highlights the increased randomness of the system during the adsorption process.^97^

The endothermic nature of the adsorption reveals that the adsorption capacity of the beads increases with temperature.^98,99^ However, the increase in the adsorption capacity with temperature is limited, a result which is compatible with the results obtained from PBD where the factor did not show a significant effect on adsorption. As Δ*H*° is lower than 20.9 kJ mol^–1^, the process can be considered as physical adsorption,^100−102^ which is consistent with the results obtained from the D–R isotherm model.

### 3.7 Statistical Design of Experiment and Optimization of Recovery Variables Using CCD

RSM with CCD design was performed to obtain the optimum values of the two independent parameters of the recovery process. The effects of the HNO_3_ concentration and time were studied. Eleven experiments with three replicates and three center points were performed. The obtained experimental and predicted values of CCD recovery are listed in Table 7.

**Table 7:** Experimental Design According to CCD and Experimental and Predicted Values of Recovery (%) (Number of Independent Parameters: 2; Number of Runs: 11; and Number of Center Points: 3)

			recovery	
run	conc. of HNO_3_ (M)	time (min)	experimental (%)	predicted (%)
1	0.095	25.000	22.0	22.29
2	1.000	10.000	52.0	60.67
3	1.155	25.000	97.7	89.34
4	0.625	25.000	11.4	10.67
5	0.625	25.000	14.0	10.67
6	0.250	10.000	7.4	9.95
7	0.250	40.000	58.0	57.41
8	0.625	25.000	6.6	10.67
9	0.625	46.213	92.0	90.19
10	0.625	3.786	34.0	27.74
11	1.000	40.000	96.0	100.00

The ANOVA results for the quadratic model employing a second-order polynomial with the RSM using CCD are presented in Table 8. The model was statistically significant. The *R*^2^, adjusted *R*^2^, and predicted *R*^2^ values for this model were 0.9812, 0.9623, and 0.8762, respectively.

**Table 8:** ANOVA for RSM Using CCD for the Recovery Process^a^

source	sum of squares	degree of freedom	mean square	*F*-value	*p*-value	
model	13179.06	5	2635.81	52.0600	0.0003	significant
A	4496.17	1	4496.17	88.8100	0.0002	
B	3899.52	1	3899.52	77.0300	0.0003	
AB	10.89	1	10.89	0.2151	0.6623	
A^2^	2877.38	1	2877.38	56.8400	0.0007	
B^2^	3292.92	1	3292.92	65.0400	0.0005	
residual	253.13	5	50.63			
lack of fit	224.94	3	74.98	5.3200	0.1623	not significant
pure error	28.19	2	14.09			
total	13432.19	10				

a *R*^2^ = 0.9812; adjusted *R*^2^ = 0.9623; and predicted *R*^2^ = 0.8762.

The final equation obtained in terms of the coded factors is as follows
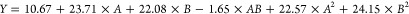
6where *Y* is the response (recovery, %), *A* is the coefficient of the HNO_3_ concentration, and *B* is the coefficient of time.

**Figure 7:**
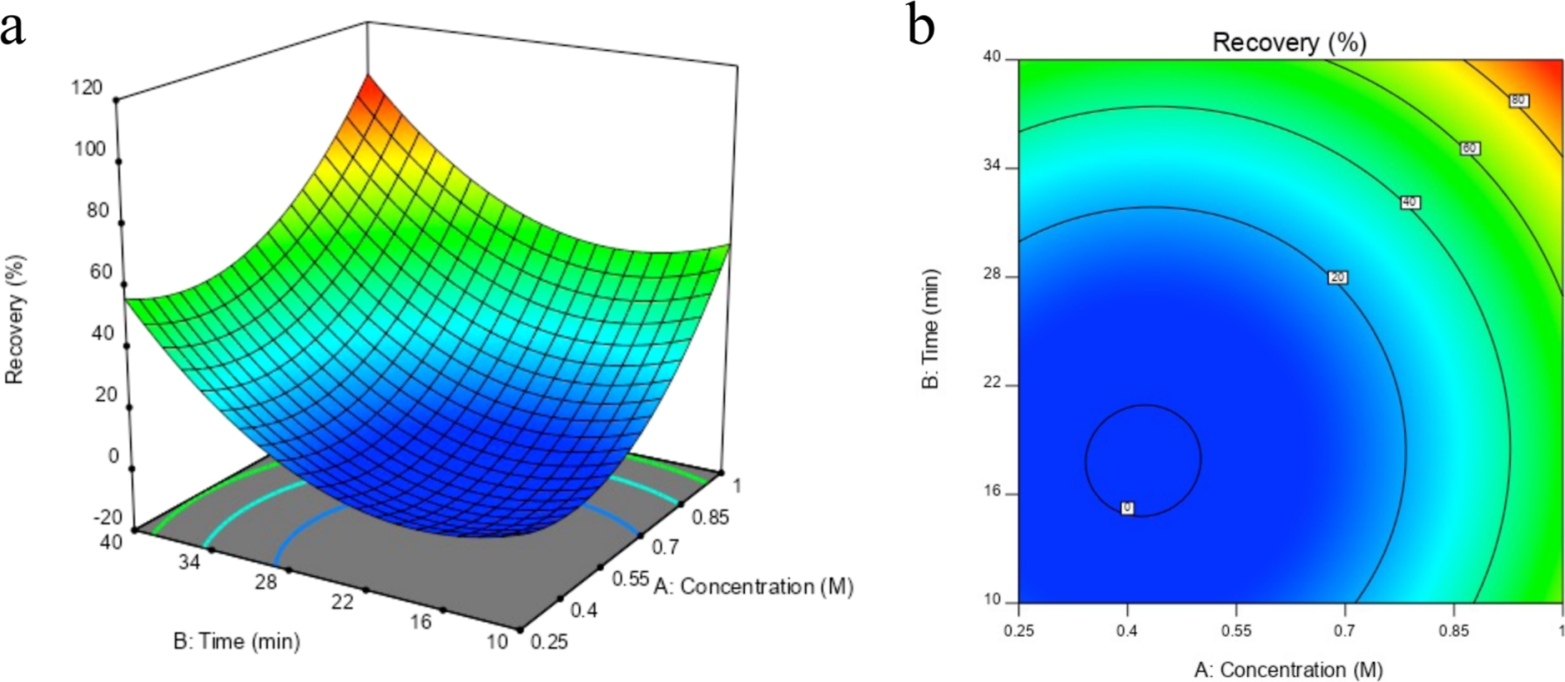
(a) 3D response surface plot and (b) contour plot of the desorption as a function of two independent variables: time and the concentration of HNO_3_ (recovery time: 0–40 min and concentration of HNO_3_: 0.25–1 M).

It can be clearly seen in the 3D response surface plot and recovery contour plot (Figure 7a) that there is only one solution for quantitative recovery. The maximum recovery was obtained at the point where the HNO_3_ concentration was 1 mol L^–1^ and the time was 40 min (Figure 7b). These points were shown to be the optimum values in the confirmation experiments (Table S7).

### 3.8 Effect of Adsorbent Dose

The adsorbent dose is a significant parameter that affects the adsorption efficiency. Thus, it is important to determine the minimum adsorbent dose required for the quantitative adsorption of aluminum. As shown in Figure S7, a minimum dose of 30 g L^–1^ was used to ensure the quantitative adsorption of aluminum. At doses below 30 g L^–1^, the number of active exchangeable sites was insufficient for quantitative adsorption. As the number of beads increased, the number of active exchangeable sites also increased, thereby increasing the adsorption efficiency. Beyond the selected dosage, the sorbent reached its maximum capacity, and no further increase was observed in the adsorption efficiency.

### 3.9 Effect of Inorganic Ions

The interference effects of other cations, such as Na^+^, K^+^, Mg^2+^, Ca^2+^, Cu^2+^, Fe^2+^, Mn^2+^, Zn^2+^, Pb^2+^, and Fe^3+^ and anions, such as Cl^–^, NO_3_^–^, and SO_4_^2–^, on aluminum detection were investigated. The interference effect was studied independently for each ion. The adsorption, recovery, and fluorimetric determination procedures were carried out as described above in a binary mixture composed of 5 mL of 100 μg L^–1^ Al^3+^ solution (pH 4.5) and different concentrations of the ions.

The tolerable concentration ratios of the investigated ions that did not exhibit any remarkable change (>± 5%) in the recovery of aluminum were determined and are shown in Table S8.

Also, as stated in the literature, the significant interference effects of F^–^ and PO_4_^3–^ can be prevented with acid digestion,^37,103^ and Fe^3+^ can be eliminated with the addition of 10% (w/v) l-ascorbic acid to the sample solution before the fluorimetric determination procedure.^104^

### 3.10 Reusability of Beads

The reusability of the magnetic beads, both with and without GA cross-linking, was investigated. When magnetic beads without GA cross-linking were used, though the quantitative adsorption (98.1% ± 0.2%) and recovery (98.0% ± 1.2%) of aluminum after four cycles (*n* = 4) were high, the acid was found to damage the beads and magnetic nanoparticles were observed in the solution. In contrast, the quantitative adsorption (98.2% ± 0.3%) and recovery of aluminum (99.4% ± 2.6%) were high after 10 cycles (*n* = 10) for GA cross-linked magnetic beads, and no release of the magnetic nanoparticles into the solution was observed, thus confirming the importance of cross-linking. The significant increase in the number of cycles can be attributed to the enhanced mechanical strength and stability of the hydrogel cross-linked with GA.

### 3.11 Analytical Figures of Merit

The calibration graph was linear in the range of 0.02–2 mg L^–1^ Al(III), which was suitable for the analysis of trace levels of the analyte. The calibration graph had an equation of *y* = 6818.3*x* + 14.422 and an *R*^2^ of 0.9973 (*n* = 3). The limit of detection (LOD) and limit of quantification were calculated as 4.3 and 14 μg L^–1^, respectively, based on the equations given in the Supporting Information.

The general adsorption parameters of the various adsorbents used for the determination of aluminum are given in Table 9. Specifically, considering the capacity of the adsorbent and the LOD, the method developed in this study, based on the use of magnetic hydrogel beads, could be useful for the analysis of trace levels of aluminum in real samples.

**Table 9:** General Adsorption Parameters of the Adsorbents Used for the Determination of Aluminum

adsorbent/detection	capacity (mg g^–1^)	kinetic model	adsorption isotherm model	reference
gellan gum, acrylic acid double network/AAS	13.50	PSO	Langmuir	(105)
ion-imprinted polymer/ICP-OES	106.00	PSO	Langmuir	(106)
S957 chelation resin/AAS	40.00	PFO		(107)
modified rice husk powder/spectrophotometry	2.87	PSO	Langmuir	(108)
K10, TiO_2_, SiO_2_/ICP-OES	0.96 (K10)	PSO (K10)	Freundlich (TiO_2_ and SiO_2_)	(109)
	0.45 (TiO_2_)	PFO (SiO_2_)	Langmuir and Sips (K10)	
	0.53 (SiO_2_)	PFO (TiO_2_)		
magnetic hydrogel bead/fluorescence	5.25	PSO	Langmuir	this study

### 3.12 Sample Application

The proposed method was used to determine the amount of aluminum in tap water and carboy water samples. Water samples were first filtered using a cellulose acetate filter with a pore size of 0.45 μm, and then the pH of the samples was adjusted to 4.5. The water sample (10 mL) was shaken with 1200 mg of GA-cross-linked magnetic hydrogel beads for 30 min. After the adsorption of Al(III), the adsorbent was separated from the solution using a magnet. Subsequently, 5 mL of 1 M HNO_3_ was added to the adsorbent and shaken for 40 min for aluminum recovery. The spike addition method was used for three different concentrations and three parallel analyses. Quantitative recoveries were obtained, and the results obtained by using the proposed method were compared with those obtained by using ICP-OES. The results are presented in Table 10.

**Table 10:** Analytical Application of the Developed Method (pH: 4.5; Sample Volume: 10 mL; Adsorbent Amount: 1200 Mg; Contact Time for Adsorption: 30 min; Recovery Agent: 1 M HNO_3_; and Recovery Time: 40 min)

sample	added Al(III) (μg/L)	Al(III) detected with the developed method (μg/L)	Al(III) detected with ICP-OES (μg/L)
tap water	<LOD^a^		
	100	103 ± 10	99 ± 4
	250	248 ± 12	243 ± 4
	500	489 ± 17	506 ± 25
carboy water	<LOD^a^		
	100	107 ± 8	98 ± 3
	250	256 ± 10	253 ± 8
	500	501 ± 38	508 ± 10

a LOD = limit of detection (*n* = 3).

## 4 Conclusions

This paper proposes, for the first time, the use of magnetic hydrogel beads as adsorbents for the detection of aluminum. The adsorption and recovery process parameters were optimized by using statistical experimental designs. The adsorbent was fully characterized and reused for 10 successive adsorption–desorption cycles. The aluminum detection was completed within 70 min. Thermodynamic studies revealed that the adsorption was spontaneous and endothermic. The pseudo-second-order model fitted the adsorption process well, thereby indicating that the adsorption rate was controlled by chemisorption. The evaluation of the isotherm models confirmed that aluminum adsorption on the beads was monolayer and physical adsorption on a homogeneous surface with an adsorption capacity of 5.25 mg g^–1^. The developed method was applied to tap water and carboy water samples, and the results showed that magnetic hydrogel beads can be a useful method for the detection of trace levels of aluminum in aqueous samples.

## Supporting Information Available

Illustration of bead synthesis; EDX analyses; SEM images; VSM analyses; DLS analysis; stress/strain curves; adsorbent dose effect; effect of reagents on recovery; details of CCD; BBD confirmatory experiments; kinetic studies; isotherm models; thermodynamic parameters; CCD confirmatory experiments; and effects of inorganic ions (PDF)
