# Supplementary material for: Magnetic Hydrogel Beads as a Reusable Adsorbent for Highly Efficient and Rapid Removal of Aluminum: Characterization, Response Surface Methodology Optimization, and Evaluation of Isotherms, Kinetics, and Thermodynamic Studies
Source: ACS Omega. 2023 Nov 3;8(45):42440–56. doi: 10.1021/acsomega.3c04984 (PMC10652826; doi:10.1021/acsomega.3c04984)
Supplement: Supplementary file 1 — ao3c04984_si_001.pdf [file ao3c04984_si_001.pdf]

SUPPORTING INFORMATION

Magnetic Hydrogel Beads as a Reusable Adsorbent for Highly Efficient and Rapid Removal of Aluminum: Characterization, Response Surface Methodology Optimization and Evaluation of Isotherms, Kinetics and Thermodynamic Studies

Raif İLKTAÇ[1,*] and Ece BAYIR[1]

[1] Ege University Central Research Test and Analysis Laboratory Application and Research Center (EGE-MATAL), Ege University, 35100, Izmir, Turkey

The equations of the pseudo-first order, pseudo-second order and intra-particle diffusion models can be shown as follows, respectively:

$$\log (q_e - q_t) = \log q_e - \frac{k_1}{2.303}t \qquad \text{(Equation S1)}$$

$$\frac{t}{q_t} = \frac{1}{k_2 {q_e}^2} + \frac{t}{q_e} \qquad \text{(Equation S2)}$$

$$q_t = k_{id}t^{\frac{1}{2}} + C \qquad \text{(Equation S3)}$$

where q_e is the amount of the adsorbed aluminum at equilibrium (mg g^{-1}), q_t is the amount of aluminum adsorbed at time t (mg g^{-1}), k_1 is the pseudo first order rate constant (min^{-1}), k_2 is the pseudo second order rate constant (g mg^{-1} min^{-1}) and k_{id} is the intraparticle diffusion rate constant (mg g^{-1} $min^{-1/2}$), t is the time (min) and C (mg g^{-1}) represents the boundary layer thickness.

The equations of Freundlich, Langmuir and Dubinin–Radushkevich (D-R) isotherm models can be shown as follows, respectively:

$$\ln q_e = \ln K_F + \frac{1}{n} lnC_e \qquad \text{(Equation S4)}$$

$$\frac{C_e}{q_e} = \frac{1}{K_L Q_m} + \frac{C_e}{Q_m} \qquad \text{(Equation S5)}$$

$$\ln q_e = \ln q_m - k\varepsilon^2 \text{ and } E = (2k)^{-0.5} \qquad \text{(Equation S6)}$$

In Freundlich isotherm model, q_e is the amount of aluminum adsorbed by the adsorbent (mg g^{-1}), C_e is the equilibrium concentration of aluminum (mg L^{-1}), K_F (mg g^{-1}) and n (dimensionless) are Freundlich constants related to the adsorption capacity and intensity of adsorption, respectively. Freundlich isotherm was obtained by plotting ln C_e versus ln q_e.

In Langmuir isotherm model, C_e is the equilibrium concentration of aluminum (mg L^{-1}), q_e is the adsorption capacity at equilibrium (mg g^{-1}), Q_m is maximum adsorption capacity (mg g^{-1}) and K_L is the Langmuir adsorption constant (L mg^{-1}). Langmuir isotherm was obtained by plotting Ce/qe versus Ce.

For D-R isotherm model, q_e is the amount of aluminum adsorbed by the adsorbent (mol g^{-1}), q_m is the maximum sorption capacity (mol g^{-1}), k is the activity coefficient related to sorption energy (mol^2 kJ^{-2}), R is the gas constant (J $mol^{-1}K^{-1}$), T is the temperature (K), ε is the Polanyi potential (J mol^{-1}), C_e is the equilibrium concentration of aluminum (mol L^{-1}) and E is the sorption energy represents the energy required for moving one mole of the solute from infinity to the surface of the adsorbent (kJ mol^{-1}). D-R isotherm was obtained by plotting ln q_e versus ε^2. Parameters of the equations were calculated using the slope and intercept of the plots.

Gibbs free energy change (ΔG°), enthalpy change (ΔH°) and entropy change (ΔS°) were calculated from the following equations:

$$\Delta G^0 = -RTlnK_C\,,\ K_C = \frac{C_S}{C_e} \qquad \text{(Equation S7)}$$

$$lnK_C = \frac{\Delta S^0}{R} - \frac{\Delta H^0}{RT} \qquad \text{(Equation S8)}$$

$$\Delta G^0 = \Delta H^0 - T\Delta S^0 \qquad \text{(Equation S9)}$$

where K_c is the equilibrium constant, C_s is the amount of aluminum adsorbed by adsorbent (mg g^{-1}), C_e is the equilibrium concentration of aluminum (mg L^{-1}), R is the gas constant (8.314 J mol^{-1} K^{-1}) and T is the temperature (K).

Limit of detection (LOD) and limit of quantification (LOQ) values were calculated using the equations;

$$\text{LOD} = \frac{\sigma}{S} \times 3 \qquad \text{(Equation S10)}$$

$$\text{LOQ} = \frac{\sigma}{S} \times 10 \qquad \text{(Equation S11)}$$

where σ is the standard deviation of the responses of blank solution and S is the slope of the calibration curve.

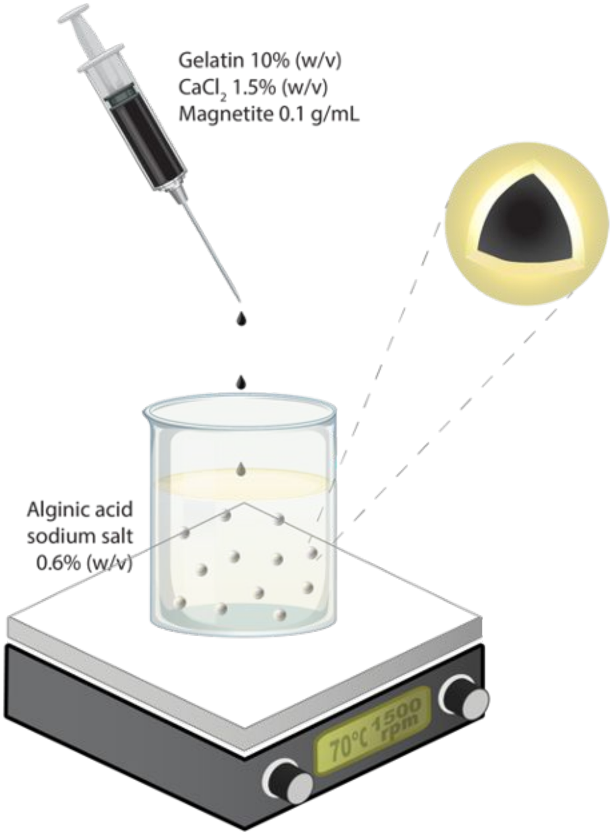

Figure S1. Illustration of hydrogel bead synthesis (temperature: 70°C, agitation speed: 1500 rpm).

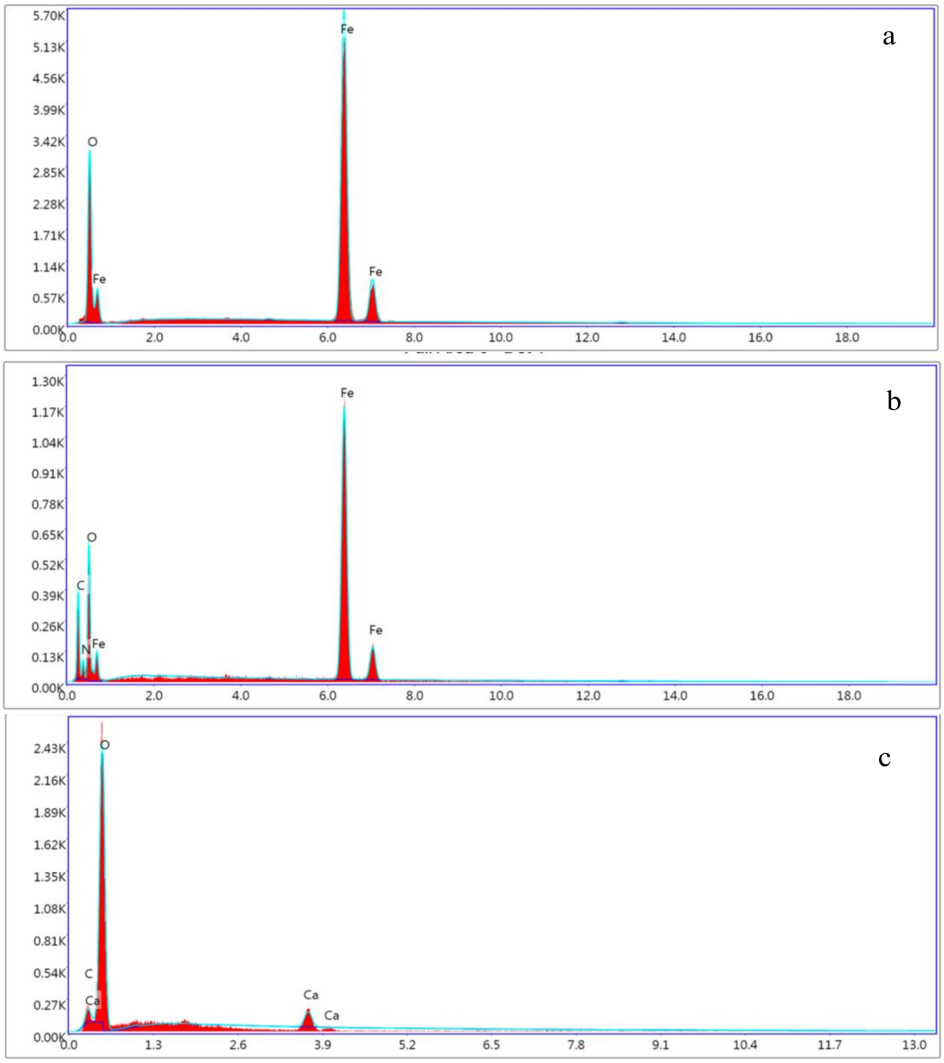

Figure S2. EDX analysis of a) magnetite nanoparticles b) cross-section of the beads and c) outer surface of the beads (accelerating voltage: 30.0 kV, working distance: 10 mm).

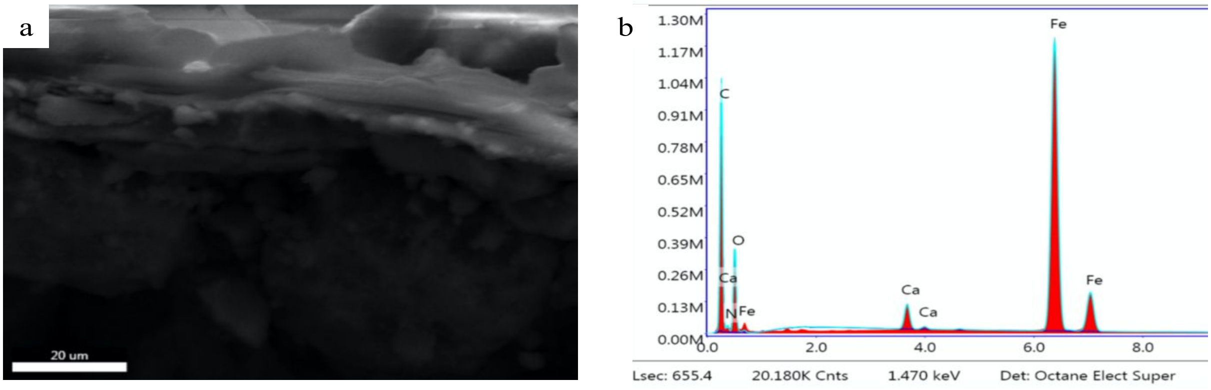

Figure S3. a) SEM image and b) EDX spectrum of the related cross-section (accelerating voltage: 30.0 kV, working distance: 10 mm, magnification: x3500).

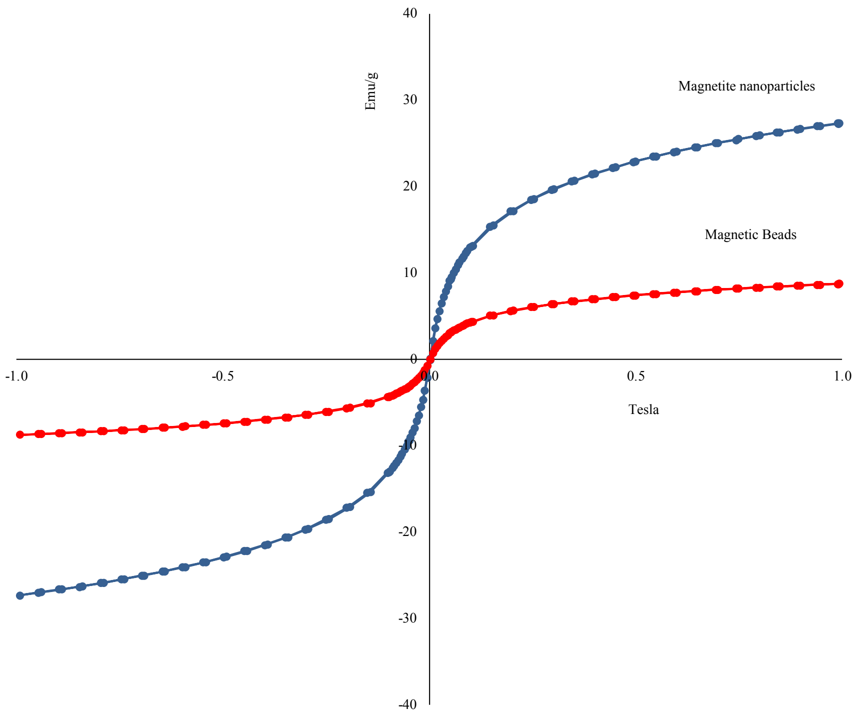

Figure S4. VSM analysis of magnetite nanoparticles and Fe3O4@glu-GA beads (temperature: room temperature, applied magnetic field: -1 to 1 Tesla).

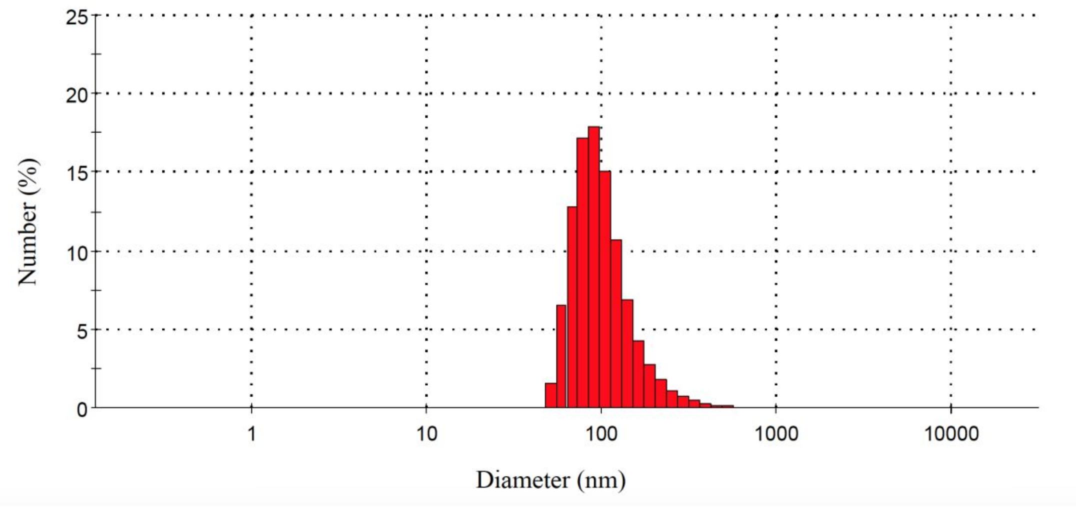

Figure S5. Size distribution graphic of magnetite nanoparticles determined by DLS (temperature: 25°C, concentration of nanoparticles: 0.1 mg mL^{-1} in UPW).

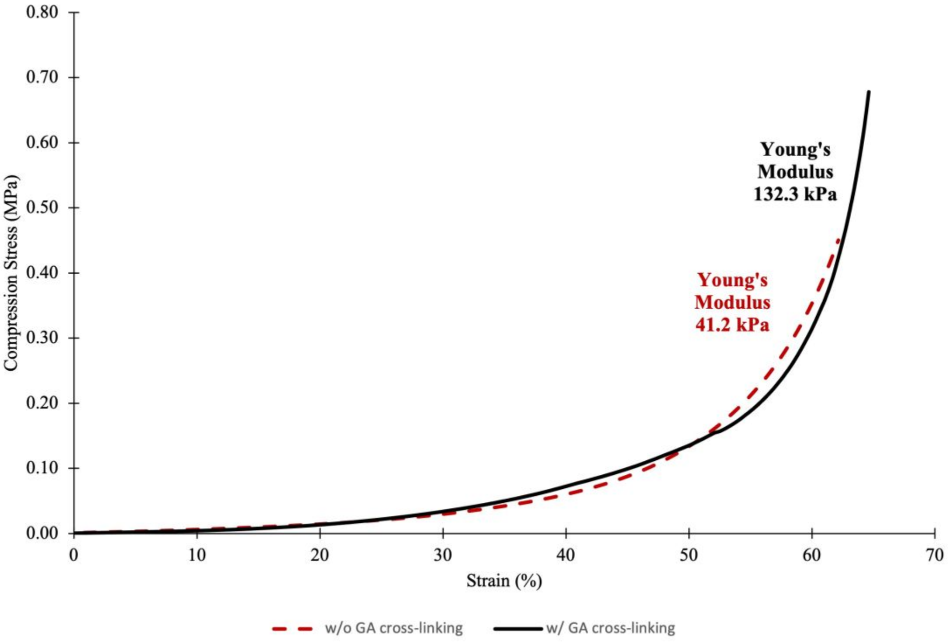

Figure S6. The uniaxial compression stress-strain curves of beads with (black straight line) and without GA cross-linking (red dashed line). Young's Moduli of the beads were represented on curve (diameter of samples: 3.82 ± 0.04 mm, thickness: 5.67 ± 0.8 mm, ramp force: 0.5 N/min, temperature: 25°C).

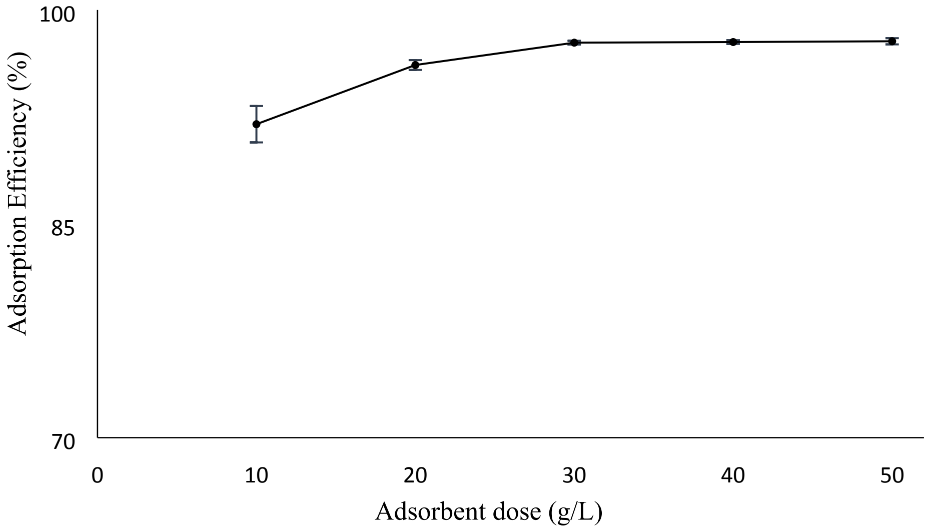

Figure S7. The effect of adsorbent dose (pH: 4.5, concentration of Al(III): 1 mg L^{-1}, temperature: 25°C, contact time: 30 min, agitation speed: 150 rpm).

Table S1. Effect of type and concentration of reagent on recovery of aluminum (concentration of reagent: 1 mol L^{-1}, volume: 5 mL, recovery time: 60 min, agitation speed: 150 rpm).

**Reagent**	**Volume (mL)**	**Recovery (%) (n=3)**
HNO_3 (1 mol L^{-1})	5.0	98.1 ± 3.8
HCl (1 mol L^{-1})	5.0	Destructed bead
CH_3COOH (1 mol L^{-1})	5.0	47.0 ± 5.7
NaOH (1 mol L^{-1})	5.0	3.9 ± 1.2

Table S2. Optimization parameters and levels of CCD (reagent: HNO_3, number of independent variables: 2).

		**Levels**				
**Factor**	**Factor Code**	$-\alpha$	**Low**	**Center**	**High**	$+\alpha$
Concentration of HNO_3 (M)	A	0.095	0.25	0.625	1	1.155
Adsorption time (min)	B	3.786	10	25	40	46.213
α = 1.41421						

Table S3. Confirmatory experiments for BBD matrix model validation and confirmation (pH: 4-4.5, amount of adsorbent: 488-600 mg, contact time: 28.7-30 min, number of solutions: 3).

				**Adsorption Efficiency**	
**Solution**	**pH**	**Amount (mg)**	**Time (min)**	**Experimental (%)**	**Predicted (%)**
1	4.5	600.0	30.0	99.4	100.0
2	4	526.0	31.5	97.8	97.9
3	4.5	488.0	28.7	97.2	96.3

Table S4. Kinetic parameters of different models of aluminum sorption (sample volume: 5 mL, concentration of Al(III): 20 mg L^{-1}, pH: 4.5, amount of adsorbent: 100 mg, contact time: 5-1440 min, temperature: 25 °C, agitation speed: 150 rpm).

Kinetic model	Parameters	Plot
pseudo-first order	q_e = 1.3 mg g^{-1}<br>k_1 = 0.014 min^{-1}<br>R^2 = 0.9633	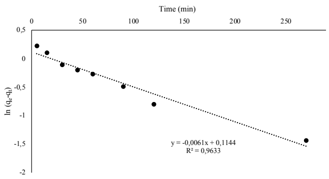
pseudo-second order	qe= 2.6 mg g^{-1}<br>k_2 = 0.024 g mg^{-1} min^{-1}<br>R^2 = 0.9990	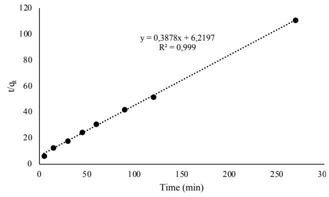
intra-particle diffusion	C = 0.6 mg g^{-1}<br>k_{id} = 0.1691 mg g^{-1} $min^{-1/2}$<br>R^2 = 0.9456	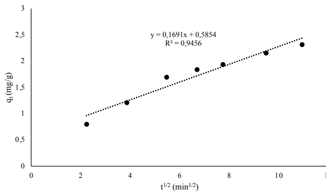

Table S5. Sorption isotherm models (sample volume: 5 mL, amount of adsorbent: 200 mg, initial Al(III) concentration: 1-50 mg L^{-1}, pH: 4.5 contact time: 24 h, temperature: 25 °C, agitation speed: 150 rpm).

Model	Parameters	Plot
Freundlich	$K_F =$ 3.28 mg g^{-1}<br>n = 1.49<br>R^2 = 0.9385	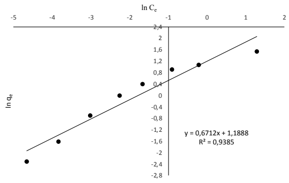
Langmuir	$Q_m =$ 5.25 mg g^{-1}<br>$K_L =$ 1.99 L mg^{-1}<br>R^2 = 0.9973<br>R_L=0.01	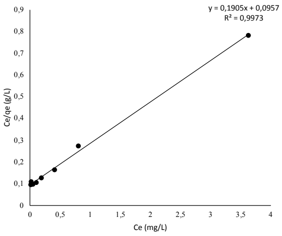
Dubinin–Radushkevich	k = 0.028 mol^2 kJ^{-2}<br>q_m = 0.00012 mol g^{-1}<br>E = 4.23 kJ mol^{-1}<br>R^2 = 0.9681	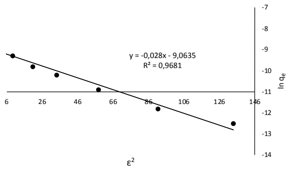

Table S6. Thermodynamic parameters of aluminum sorption (temperature: 25- 45°C, sample volume: 5 mL, concentration of Al(III): 20 mg L^{-1}, pH: 4.5, amount of adsorbent: 100 mg, time: 24h, agitation speed: 150 rpm).

**Temperature / K**	**ΔG° / kJ mol^{-1}**	**ΔH° / kJ mol^{-1}**	**ΔS° / kJ mol^{-1} K^{-1}**
298	-22.94	13.27	0.12
308	-24.16		
318	-25.37		

Table S7. Confirmatory experiments for CCD matrix model validation and confirmation (number of solutions: 3, concentration of HNO_3: 0.992-0.997 mol L^{-1}, time: 35-40 min).

			**Recovery**	
**Solution**	**Conc. of HNO_3 (M)**	**Time (min)**	**Experimental (%)**	**Predicted (%)**
1	0.997	39.99	99.8	100
2	0.900	39.95	87.3	85
3	0.992	35.01	79.1	80

Table S8. Effects of inorganic ions on recovery of aluminum (sample volume: 5 mL, Al(III) concentration: 100 µg L^{-1}, sorption contact time: 30 min, pH: 4.5, recovery agent: 1 M HNO_3, recovery time: 40 min, temperature: 25 °C, agitation speed: 150 rpm).

**Interfering Ion**	**Tolerable Concentration Ratio**	**Recovery (%) ***
Na^{+}	1000	97.8+2.2
K^{+}	1000	98.6+1.1
Mg^{2+}	250	94.0+1.1
Ca^{2+}	1000	101.0+3.1
Cu^{2+}	100	93.8+2.6
Fe^{2+}	250	93.5+1.9
Mn^{2+}	750	98.9+4.3
Zn^{2+}	1000	101.2+2.2
Pb^{2+}	500	95.8+3.0
Fe^{3+}	100	98.9+4.3**
Cl^{-}	1000	98.5+3.7
NO_3^{-}	1000	94.7+0.3
SO_4^{2-}	1000	96.7+5.2

*(n=3)

**In the presence of 10% L-ascorbic acid
